# Design and Impact: Navigating the Electrochemical Characterization Methods for Supported Catalysts

**DOI:** 10.1021/acscatal.4c03271

**Published:** 2024-07-25

**Authors:** Karl-Ander Kasuk, Jaak Nerut, Vitali Grozovski, Enn Lust, Anthony Kucernak

**Affiliations:** † Institute of Chemistry, University of Tartu, Ravila 14a, 50411 Tartu, Estonia; ‡ Department of Chemistry, Imperial College London, 80 Wood Lane, W12 7TA London, United Kingdom

**Keywords:** Oxygen Reduction Reaction, Rotating Disk Electrode, Floating Electrode, Gas Diffusion Electrode, Membrane Electrode Assembly, Ultramicroelectrode

## Abstract

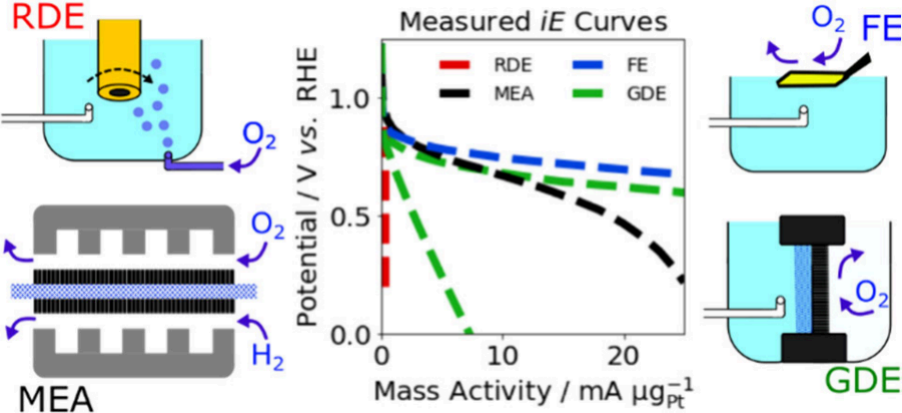

This review will investigate the impact of electrochemical characterization method design choices on intrinsic catalyst activity measurements by predominantly using the oxygen reduction reaction (ORR) on supported catalysts as a model reaction. The wider use of hydrogen for transportation or electrical grid stabilization requires improvements in proton exchange membrane fuel cell (PEMFC) performance. One of the areas for improvement is the (ORR) catalyst efficiency and durability. Research and development of the traditional platinum-based catalysts have commonly been performed using rotating disk electrodes (RDE), rotating ring disk electrodes (RRDE), and membrane electrode assemblies (MEAs). However, the mass transport conditions of RDE and RRDE limit their usefulness in characterizing supported catalysts at high current densities, and MEA characterizations can be complex, lengthy, and costly. Ultramicroelectrode with a catalyst-filled cavity addresses some of these problems, but with limited success. Due to the properties discussed in this review, the recent floating electrode (FE) and the gas diffusion electrode (GDE) methods offer additional capabilities in the electrochemical characterization process. With the FE technique, the intrinsic activity of catalysts for ORR can be investigated, leading to a better understanding of the ORR mechanism through more reliable experimental data from application-relevant high-mass transport conditions. The GDEs are helpful bridging tools between RDE and MEA experiments, simplifying the fuel cell and electrolyzer manufacturing and operating optimization process.

## 1 Introduction

Electrolyzers and fuel cells can be used to stabilize the intermittency of the renewable energy-based electricity grid. Hydrogen can be produced with electrolyzers when there is too much renewable electricity production and turned back to electricity using fuel cells when needed.^1^ Additionally, hydrogen fuel cells can be used to power vehicles, off-grid locations, or hospitals in case of power failure without emitting any dangerous emissions. One promising technology for the mentioned uses is the proton exchange membrane (PEM) fuel cell (FC) technology.^1^

Increasing the efficiency and lifetime of PEMFC has been a perennial problem.^2^ The two reactions in the PEMFC are the hydrogen oxidation reaction (HOR) (eq 1) and the oxygen reduction reaction (ORR) (eq 2). The reverse reactions—hydrogen evolution reaction (HER) and oxygen evolution reaction (OER)—occur in the PEM electrolyzers which are one of the promising options for producing green hydrogen from water and renewable electricity. Catalysts, usually based on platinum group metals (PGMs), are used to reduce the overpotential and thus increase the efficiency of the reactions occurring in the PEMFC and electrolyzers.
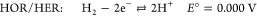
1
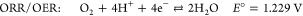
2

For a typical hydrogen-powered vehicle, Toyota Mirai, the PEMFC anode, where the HOR takes place, requires 0.05 mg cm^−2^ of platinum, and the cathode, where the ORR occurs, must have 0.315 mg cm^−2^ of platinum with a total amount of 22–36 g of platinum per vehicle.^2,3^ Thus, much effort is invested in reducing or removing the platinum and other PGM content. Non-PGM catalysts are in the development stage but have yet to reach the targets for broad commercialization.^2^

The PGM loading for electrolyzers is even higher. However, as with PEMFC, the hydrogen side in the PEM electrolyzer is less of a problem. The cathode, where the HER takes place, commonly uses about 1.0 mg cm^−2^ of platinum-based catalysts.^4^ On the other hand, the OER on the anode requires around 2.0 mg cm^−2^ of iridium- or ruthenium-based oxide as a catalyst due to the highly oxidative environment.^4^ Moreover, noble metal coatings are sometimes used on the PEM electrolyzer bipolar plates, which further increases the overall PGM mass in a stack.^5^

**Figure 1:**
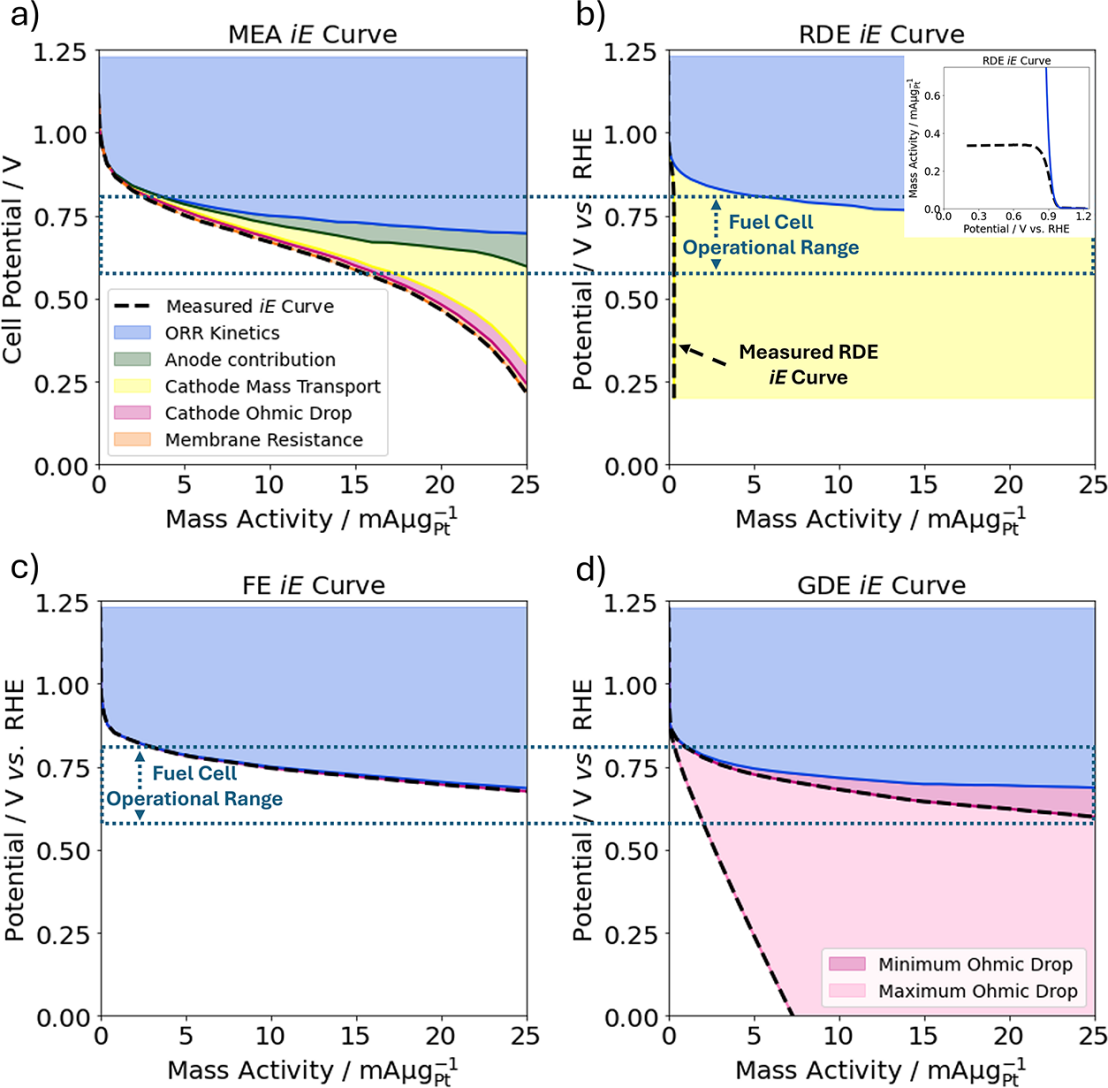
Illustrative schematics of common oxygen reduction reaction (ORR) *iE* curves of (a) membrane electrode assembly (MEA) showing contributions from ORR kinetics calculated using the Koutecký-Levich (K-L) model, anode kinetics and mass transport as anode contribution, cathode mass transport including flooding, cathode ohmic drop, and membrane resistance;^6^ (b) rotating disk electrode (RDE) comparing a common measured *iE* curve and ORR kinetics estimation using the K-L model with an inset showing the same RDE graph in a different scale and with axis flipped; (c) floating electrode (FE) with a small *iR* correction component due to small absolute currents;^7^ and (d) gas diffusion electrode (GDE) with a minimum and maximum *iR* correction due to different setups.^8^ The proton exchange membrane fuel cell operational range of around 0.6–0.8 V vs RHE^9,10^ is indicated with a blue dotted line.

In a fuel cell or electrolyzer, the catalyst is not the only factor affecting the cell performance. Taking PEMFC as an example, as will be done in the rest of this review, one can see that the performance is broadly determined by the ORR kinetics, anode contribution, cathode mass transport, cathode ohmic drop, and membrane resistance (Figure 1a).^6^ These listed factors are determined by the different cell layers and their interfaces. However, to research and develop better fuel cells and electrolyzers, a systematic approach should be taken to understand and improve one component and one interface at a time, starting with the catalyst. Different electrochemical characterization methods should be used to determine the influence of a chosen variable on electrochemical performance.

In five sections, this review will highlight the central design principles and unintended consequences of the main electrochemical characterization methods for investigating supported ORR catalysts (as opposed to single crystal or electrodeposited systems). The bulk of the review uses ORR on supported PGM catalysts as a case study from which to draw comparisons due to the availability of data.

By the end, the reader should be able to better understand the reasons behind

- the complexity and necessity of using the membrane electrode assembly (MEA) (Figure 1a),
- the mass transport challenges of the rotating disk electrode (RDE) technique (Figure 1b) and ultramicroelectrode with a cavity (UMEC),
- the mass transport benefits, condition limitations, and unique intrinsic activity findings of the floating electrode (FE) method (Figure 1c),
- and significance of the *iR* drop when using the gas diffusion electrode (GDE) setups (Figure 1d).

### 1.1 Descriptions of Characterization Methods

**Figure 2:**
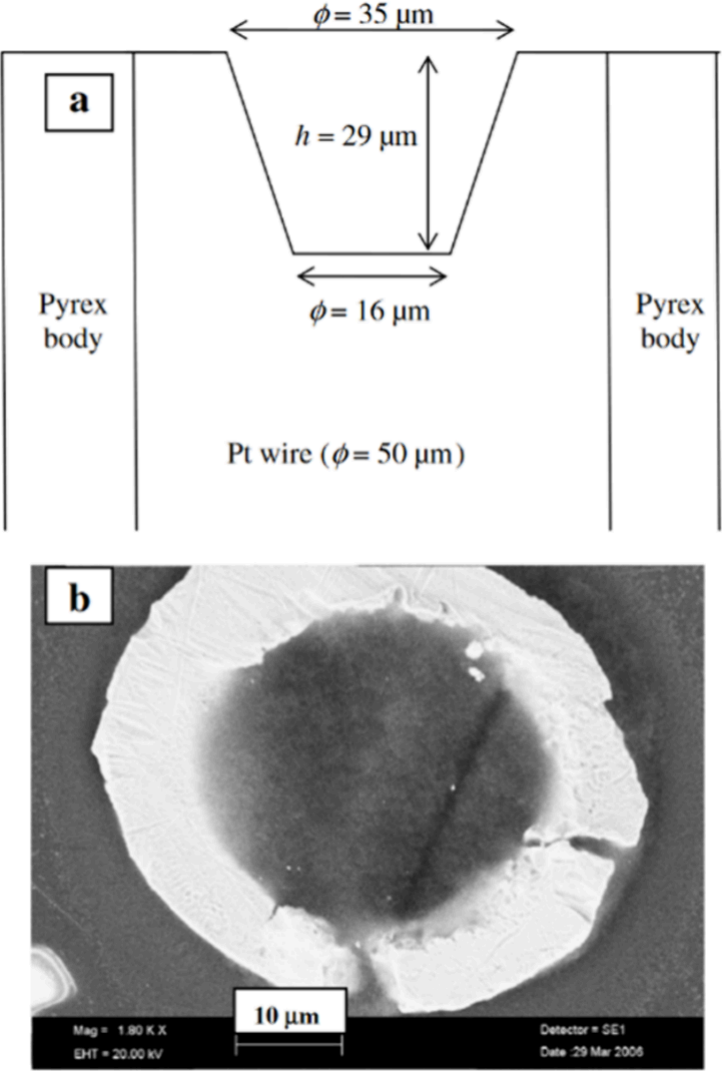
Schematic of an ultramicroelectrode with a cavity. Reproduced with permission.^14^ Copyright 2007, Elsevier.

**Figure 3:**
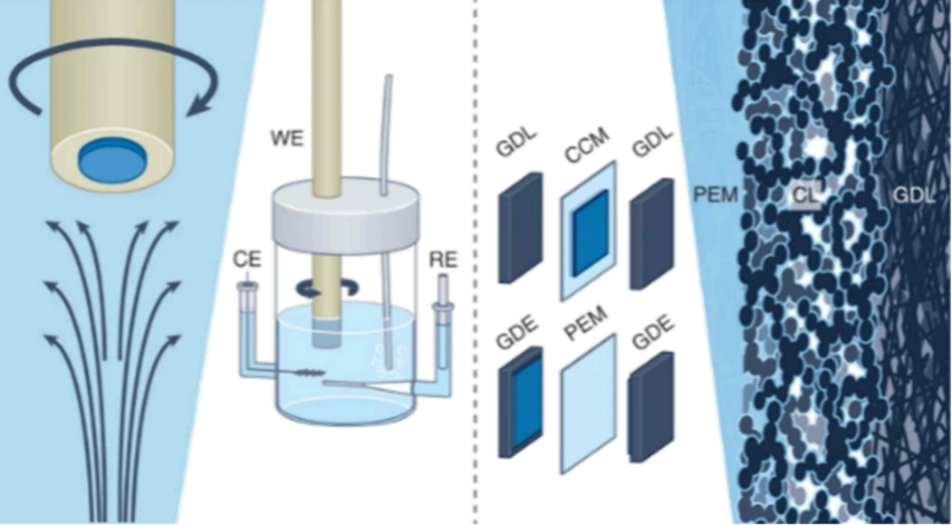
Schematics of the rotating disk electrode and the membrane electrode assembly with two construction pathways of depositing the catalyst layer (CL) on the proton exchange membrane (PEM) to make a catalyst coated membrane (CCM) or depositing the CL on a gas diffusion layer (GDL) to produce a gas diffusion electrode (GDE). Adapted with permission.^28^ Copyright 2022, Springer Nature.

**Figure 4:**
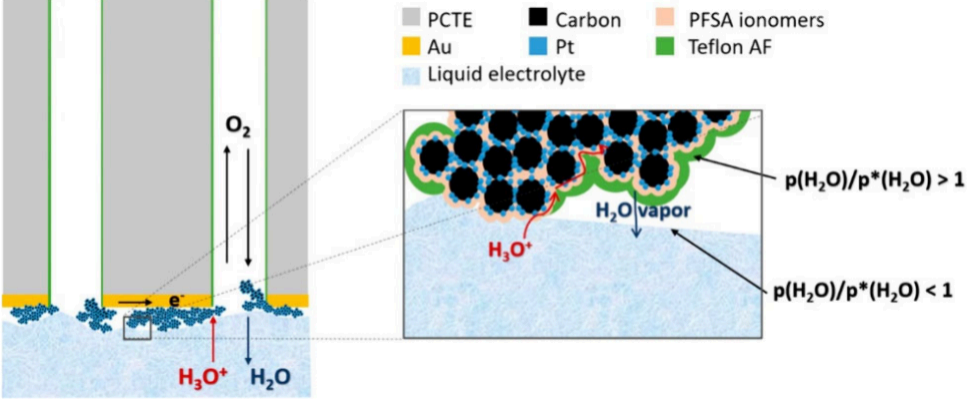
Schematic of the floating electrode setup. Reprinted with permission.^33^ Copyright 2020, American Chemical Society.

**Figure 5:**
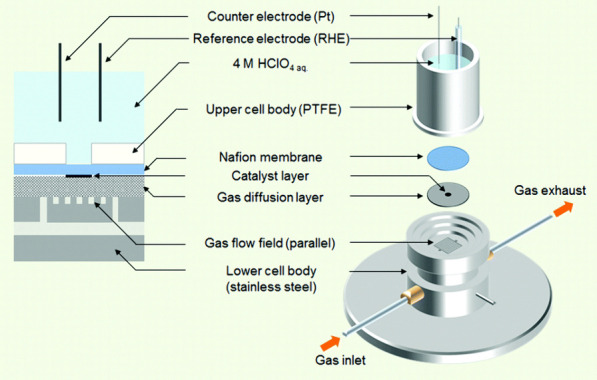
Schematic of a gas diffusion electrode characterization method. Reproduced with permission.^42^ Copyright 2008, Royal Society of Chemistry.

The five main supported catalyst electrochemical characterization methods included and characterized in this work are the ultramicroelectrode (UME) (Figure 2), RDE (Figure 3), FE (Figure 4), GDE (Figure 5), and MEA methods (Figure 3).

UMEs are a popular choice for fundamental electrochemical characterization.^11,12^ They use a micrometer-scale conductive wire inside an insulating shroud. The tip can be shaped to a desired geometry depending on the goals of the experiments.^13^ The small-size electrode allows for higher geometric current density with small absolute currents due to the higher flux caused by the hemispherical diffusion of reactants to the surface. Thus, after an initial short time period, mass transport of reactants becomes time-invariant and steady-state conditions are achieved. This contrasts with large planar electrodes where the diffusion of reactants to the surface is linear, and mass transport of reactants to the electrode constantly decreases with time. Additionally, low absolute currents reduce the potential drop in the solution phase. Popular examples include using gold, platinum or mercury as the electrode material.^12^

The size of the UME can be taken to an extreme by using nanoparticles deposited onto a single carbon fiber^15^ or even for investigating single submicrometer-sized catalyst particles electrodeposited onto carbon fiber substrates with effective electroactive radii of only a few nanometers.^16^

However, investigating separately synthesized catalyst powders requires modifying the UME and creating a cavity for the powder.^14,17,18^ A UME with a cavity (UMEC) can use powders to investigate supported catalysts such as platinum nanoparticles on carbon. It can be filled by using the electrode as a mortar in a pestle with a small quantity of catalyst material. A version of UMEC by Guilminot et al.^14^ (Figure 2) consists of a platinum wire as a contact and a substrate with a diameter of 50 μm and a cavity diameter of 35 μm.^14^ One of the central challenges with UME using nanoparticles is the accurate determination of loading because it is impossible to weigh such small amounts of catalysts.

An additional benefit of the UME for investigating nanoparticles is its use for scanning electrochemical microscopy or scanning electrochemical cell microscopy.^19^ These methods can characterize single particles of catalysts on a conductive substrate.^19^ This topic was recently reviewed by Li et al.^19^

The RDE (Figure 3) and the closely related rotating ring disk electrode (RRDE) are two of the most influential and well-established techniques for the electrochemical characterization of catalysts.^20−23^ Initially designed for model systems of flat and smooth metals or glassy carbon, they’ve been adopted for supported catalyst research. To investigate intrinsic activity and mechanisms, the ionomer and catalyst layers must be thin to reduce interlayer transport effects.^24,25^ Extensive reports outline optimal preparation and characterization protocols for this thin-film RDE (TF-RDE).^14,26,27^ Some articles argue that a well-optimized RDE testing protocol can adequately determine the activity trends seen in the MEA measurements even when the absolute values differ.^28,29^ Fundamentally, however, the catalyst conditions, such as the oxide coverage, in the RDE experiments and real operation are too different to make reliable deductions about the activity at the FC relevant potential range solely based on the RDE measurements (Figure 1b). Thus, due to its simplicity and reproducibility, using the RDE can be a useful prescreening tool for supported catalysts, but should be complemented with other electrochemical characterization setups.

Although the other hydrodynamic methods, such as the wall jet electrode^30^ and the channel flow electrode^31^ techniques, offer some unique benefits, the issues they face—mass transport and coating uniformity—are similar to those of the RDE. While these methods will not be covered explicitly in this review, the insights from this review could further aid in developing these systems.

The FE technique (Figure 4) is an alternative to the hydrodynamic methods.^32^ The FE utilizes a thin, porous polycarbonate track-etch (PCTE) membrane. On one side, the membrane is coated with gold for current collection and a vacuum-filtered catalyst (VFC) layer with a catalyst loading between 0.16 and 10 μg_Pt_ cm^–2^.^32^ The diameter of the catalyst spot is 1–2 mm but could be modified with a different VFC filter mask. The other side and the pores are coated with a thin layer of gas-permeable hydrophobic fluoropolymer to make the electrode float without being flooded. The reactant gas directly accesses the catalyst through the hydrophobic pores. Diffusion rates of reactants and products are much higher in the gas phase than in liquid, which is a considerable benefit over electrodes submerged in the electrolyte.^32^

The FE allows characterization over a broader range of potentials and achieves current densities 3 orders of magnitude higher than RDE using the same loading and type of catalyst.^32,33^ Since its development in its current form,^32^ the FE technique has been optimized, compared to RDE and MEA experiments, and used to model HOR, HER, and ORR in acidic conditions.^29,33−36^ However, interlab standardization and comparisons of results are yet to be done for the broader adoption of FE.

The MEA method is another widely used standard electrochemical characterization method (Figure 3). It is a single cell of a fuel cell or electrolyzer stack and, as such, can measure the actual performance of a catalyst in operating conditions. Generally, a proton- or hydroxide-conducting polymer electrolyte membrane is sandwiched between an anode and cathode catalyst layers. The membrane must be electrically insulating to avoid a short circuit of electrodes. These catalyst layers consist of a catalyst, an electrically conductive catalyst support and an ion-conducting polymer called ionomer. After the catalyst layer, there is an electrically conductive gas diffusion layer (GDL) onto which a hydrophobic microporous layer (MPL) is deposited. The MPL removes water generated at the catalyst layer during fuel cell operation and ensures good gas accessibility. If the catalyst layer is spray-coated onto the membrane, it is called a catalyst-coated membrane. However, the catalyst-MPL-GDL sandwich is collectively called a GDE.^28,37^ Due to its layered structure with many parts, MEA experiment components and measurement conditions for fundamental studies must be optimized for each catalyst. To measure the electrode potentials separately, research is still ongoing on incorporating a reference electrode (RE) into the MEA setup.^38−41^

The GDE as a characterization method (Figure 5) could be described as miniaturized half-cell MEAs with or without a membrane. Several GDE techniques have been published in the last two decades, including those by Chen et al.,^43^ Inaba et al.,^42,44,45^ Pinaud et al.,^46^ Ehelebe et al.,^8,9,47^ Hrnjic et al.,^48,49^ and Schmitt et al. using a cell developed by a company called Gaskatel.^50,51^ The GDE methods use a GDL with a thin catalyst layer at a gas-ionomer-catalyst interface and benefit from using less catalyst than a normal MEA. The oxidation and reduction reactions can also be investigated separately due to a half-cell three-electrode setup. These methods were recently reviewed by Loukrakpam et al.^52^ and compared in a collaborative article by Ehelebe et al.^8^

The central difference between the five reported GDE methods is the electrode geometric area, which ranges from 0.07 to 2 cm^2^. This also results in somewhat different geometries and constructions while the underlying principle of using half of an MEA setup remains the same. A recent study by Nösberger et al.^53^ made significant improvements to catalyst layer optimization to make the GDE methods more reliable for measuring intrinsic activity and as an intermediate step between RDE and MEA experiments.

## 2 Comparison of Methods: Critical Differences

### 2.1 Mass Transport

To investigate the intrinsic activity and the reaction mechanisms, variables influencing the measurable parameters—such as mass transport conditions, *iR* drop value, temperature, pressure, humidity, pH, loading, and cleanliness—must be considered. In Table 1 the differences in the electrochemical characterization method conditions are summarized. When assessing the reaction kinetics on the catalysts, particular attention should be paid to species’ mass transport to and from the catalyst layer.

**Table 1:** Comparison of Electrochemical Characterization Method Representative Conditions within Different Methods^a^

Method	UMEC	FE	RDE	GDE	MEA
Temperature/°C	≤60	≤60	≤60	≤150	≤95
Pressure/bar	1	1	1	1–4	1–4
Relative humidity/%	NA	100	NA	≤100	≤100
pH	1–14	1–10	1–14	1–14	NA
Catalyst loading/μg_Pt_ cm^–2^	800	0.5–10	4–20	5–100	5–100
Electrode area/cm^2^	0.0000096	0.008–0.0314	0.0314–0.196	0.07–2	4–100
Current^b^/A	10^–7^	0.002	0.0002	0.175	10
Ohmic resistance/Ω	10	5	20	2	0.0125
*iR* drop/V	10^–6^	0.01	0.004	0.35	0.125
Current density/A cm^–2^_Geo_	0.01	0.2	0.006	2.5	2.5

a Ultramicroelectrode with a cavity (UMEC), floating electrode (FE), rotating disk electrode (RDE), gas diffusion electrode (GDE), and membrane electrode assembly (MEA).

b In our calculations, we used the lowest electrode area for current calculation and assumed a catalyst activity under operating conditions of 5 × 10^5^ A g^–1^_Pt_ and that mass transport conditions limit UMEC and RDE maximum current.

With a reaction occurring on an electrode in an electrolyte solution during, for example, a cyclic voltammetry measurement, the concentration of reactants and products will change near the electrode surface. This will create a dynamic concentration gradient of reactive species on the electrode surface and a changing concentration profile from the bulk electrolyte to the electrode. Unless expressly addressed, this issue will prevent a steady state condition from forming. However, a steady state, where the current is independent of time, must be achieved to maintain a constant mass transport term and investigate the charge transfer kinetics. The steady-state condition means there is a fixed diffusion profile between the catalyst surface and the bulk of the electrolyte with little to no change in the concentration profile in long time scales. The latter can be achieved by, for instance, controlling the diffusion layer thickness between the bulk electrolyte and the catalyst layer in the RDE measurement.

One way to deal with this is to make the ratio of electrolyte volume to electrode surface area as large as possible. By having a very small electrode—at least one dimension smaller than a diffusion layer, usually defined to be around 25 μm^12^—and low currents, a defined hemispherical diffusion gradient will form quickly. Achieving a steady state this way is the principle behind the UME.

UMEs are used to investigate various reactions on smooth electrodes.^11^ To investigate catalyst materials other than what the electrode itself is made of, a UME with a cavity (UMEC) can be used. Guilminot et al. argued that by assuming the catalyst was entirely flooded and applying a classical macro-homogeneous model, the corrected ORR current densities measured with UMEC were comparable to TF-RDE results, like those illustrated in Figure 1b, while removing the need to make a catalyst suspension and providing lower uncertainty.^14^ However, the mass transport limitations remain as the limiting current density is about 9.6 mA cm^–2^ for the ORR.^14^ Due to the thicker catalyst layer of the UMEC, the estimated mass activity is about a third of a conventional RDE at 0.017 and 0.048 mA μg^–1^, respectively.^14^ The application-relevant mass activity starts at around 2 mA μg^–1^ (Figure 1a).

Additionally, UMEs are used to study mass transport conditions in proton exchange membranes.^54−58^ Such setups can be used to determine the diffusion coefficients and solubilities of gases and calculate the exchange current density and Tafel slope values for reactions. A review by Petrovick et al.^59^ concludes that the analytical mass transport models developed for UME with a large volume of bulk electrolyte should be replaced with numerical solutions when UME is used in contact with a relatively thin membrane.

Instead of making the electrode smaller, hydrodynamic methods are also used to achieve a steady state. Rotating the RDE and RRDE in a controlled manner creates a predictable convection that replenishes the reactants and removes the products in the electrolyte near the electrode surface. This creates a well-defined thin layer of stationary electrolyte near the electrode surface, called a Nernstian diffusion layer, where thickness is controlled by the rotation speed of the electrode.^12^ This is directly related to the limiting current density if the mass transfer step is the rate-limiting process.^12^

The simplicity of the models is the most significant benefit of the RDE and RRDE methods. Models, like the Koutecký-Levich model, are used after the measurements to separate the mass-transport effect and the kinetic activity (Figure 1b).^35,60^ However, the error value of the kinetic current density increases with the increasing contribution of mass transport effects to the measured current. The reliable region of advanced ORR models, such as the double trap model by Wang et al.^60^ with Pt-based catalysts, can be extended only to about 0.8 V vs reversible hydrogen electrode (RHE), after which it diverges from the experimental data.^35^ This is further discussed in Section 3.

**Figure 6:**
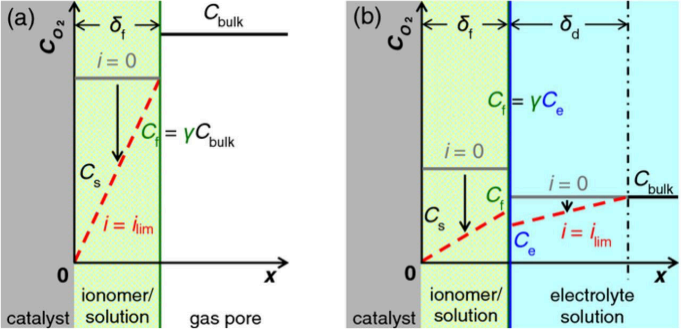
A comparison of the oxygen concentration gradient between (a) gas-accessible methods and (b) methods using dissolved gases. Reproduced under the terms of the Creative Commons Attribution 4.0 International (CC BY 4.0) license. Copyright 2015, The Electrochemical Society.^22^

The desire to extract more information from the RDE and RRDE measurements than possible leads to the misuse of these techniques.^61^ The models are based on flat and smooth electrodes, meaning the macroscopically rough (nonflat and nonuniform) catalyst layers can cause additional complications. In catalyst layers composed of high-surface-area particles, the mass transport within the catalyst layer differs from the mass transport in the electrolyte phase near the electrode surface, complicating the analysis.^42^ An ionomer layer coating the catalyst particles can also limit species’ mass transport to and from the catalyst surface (Figure 6b). Therefore, some protocols recommend removing the ionomer from the catalyst suspension and making an ionomer-free TF-RDE.^25^ The catalyst layer can also more easily cause nonlaminar flow or be removed by centrifugal forces. Thus, the rotating speeds are often limited to about 1600 rpm. This contrasts with smooth, flat RDE rotating speeds of up to 10,000 rpm (Figure 6b).^12^

RDE and RRDE are suitable for characterization at potentials down to about 0.8 V vs RHE for ORR on PGM-based catalysts. Thus, RDE can quickly filter out insufficiently active catalysts that already show low performance at low ORR overpotentials. However, these results cannot be translated to the fuel-cell-relevant potential range of about 0.6–0.8 V vs RHE^32^ as the conditions will be radically different, and the activity might not scale uniformly in a logarithmic scale as predicted by Tafel slopes plotted at low overpotentials.^62^ The mass transport limiting current densities of the RDE method in 0.1 M HClO_4_ at 1600 rpm are around 6 and 3 mA cm^–2^ for the ORR^22^ and HOR,^63^ respectively. Regardless, RDE measurement results in the small reliable current density region are sometimes extrapolated to 2 orders of magnitude higher current densities with overparametrised models.^29,34,35^

The RRDE can give interesting information about the desorbed ORR intermediates and products at the mass transport limited potentials. The ring in RRDE is mainly used to measure the hydrogen peroxide production at the disk working electrode (WE). Production of hydrogen peroxide, a two-electron process, is a competing process to four-electron ORR to water (eq 2). This can be used to make deductions about the ORR mechanism in the catalyst layer. While the usefulness of RRDE for investigating platinum catalysts for ORR is limited due to low H_2_O_2_ production on platinum catalysts, analyzing non-PGM catalysts for ORR or other reactions can provide valuable information. It must be noted that the H_2_O_2_ production can be influenced by the reactant concentration in addition to the reaction mechanisms.

RDE studies are conducted to counteract the low gas mass transport (diffusion) in the stationary electrolyte compared to mass transport in the stirred electrolyte. However, the enhancement of mass transport via rotating the electrode cannot overcome the inherent limitation of using dissolved gases because the oxygen diffusion coefficient at 20 °C is 1.9 × 10^–5^ cm^2^ s^–1^ in aqueous 0.1 M HClO_4_ solution^64^ and 0.2 cm^2^ s^–1^ in nitrogen.^32,65^ Methods with the catalyst particles at the gas | electrolyte interface (Figures 4 and 5) circumvent that issue by reducing the length of the diffusion pathway through the electrolyte to a couple of nanometers of electrolyte or ionomer coating the catalyst. This means that the mass transport of gaseous reactants mainly occurs in the gas phase, and although the diffusion in the ionomer is slow, the layer is thin enough not to limit the current density (Figure 6).^22^ This makes characterization at relevant potentials and current densities more applicable to actual PEMFC conditions where the mass transport also happens mainly in the gas phase.

The FE is one such method where the species’ transport occurs in the gas phase (Figure 4). The convection of the bulk gas phase—caused by the gas inlet—occurs until the pores of the PCTE substrate. From there to the catalyst, the transport is mainly governed by diffusion. The mass transport rate of reactants through the PCTE pores is high enough not to limit the reaction rate (Figure 1c). This means that modeling mass transport can be avoided, and analyzing intrinsic activity is trivial, *i*.*e*. the measured current density is equal to ORR kinetic current density.

**Figure 7:**
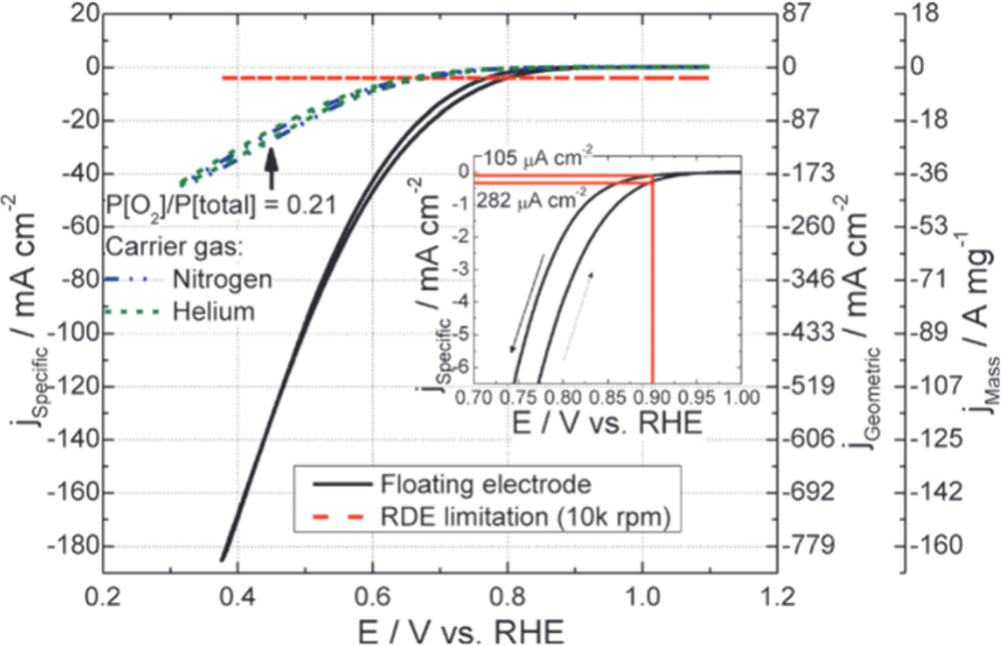
Oxygen reduction reaction with the floating electrode in a pure oxygen environment (solid black line) and in mixed O_2_–N_2_ (dash-dotted blue line) and O_2_–He (short dash green line) conditions. The rotating disk electrode (RDE) at 10,000 rpm limitation of 14 mA cm^–2^_Geo_ is shown with a dashed red line. The inset shows the hysteresis due to OH_ads_ adsorption by highlighting the ORR activity at 0.9 V vs RHE. Reprinted with permission.^32^ Copyright 2013, Royal Society of Chemistry.

Zalitis et al.^32^ tested whether the mass transport of oxygen affects the measured current density by changing the oxygen carrier gas from nitrogen to helium (Figure 7). The oxygen diffusion coefficient in helium is 0.7 cm^2^ s^–1^ at 20 °C compared to 0.2 cm^2^ s^–1^ in nitrogen.^65^ If ORR was limited by oxygen gas transport to the catalyst layer, the current density should have increased when changing the carrier gas from nitrogen to helium.^66^ However, no change in current densities was observed. Therefore, the oxygen transport to catalyst sites is not a limiting factor in the FE setup.^32^

FE high mass transport conditions allow the study of ORR at high overpotentials, and it was reported that the commercial 60% Pt/C catalyst (Alfa Aesar, HiSPEC 9100) showed equal or higher mass activity than in state-of-the-art MEA values given in literature after correcting for the higher oxygen partial pressure in FE (101 kPa) but not correcting for the lower temperature in the FE (25 °C) compared to the MEA tests (80 °C).^32^ Because catalyst activity increases with temperature, based on the FE data, the potential activity of the catalyst is not achieved in the MEA.

At higher specific current densities, measured within 0.38 to 0.6 V vs RHE, the *iE* curve follows a relatively linear line (Figure 7), which can suggest the existence of some *iR* or mass transport effects.^32^ However, the scan was curved between 0.6 and 0.8 V vs RHE (Figure 7) and thus is mainly kinetically controlled in this region of potentials. Therefore, in contrast with other characterization methods, by removing the mass transport effects that mask the catalyst activity at high current densities, the electrodes can be reliably characterized using FE at a relevant potential to the fuel cell industry of around 0.65 V vs RHE.^32^

To further validate the high mass transport conditions for FE, Zalitis et al.^67^ compared the kinetics of ORR at different catalyst loadings at 0.90 V (potential commonly used to report ORR activity in RDE measurements) and 0.65 V vs RHE (PEMFC relevant potential). For HOR kinetics, they compared current densities at 0.01 V vs RHE (potential commonly used to report HOR activity in RDE measurements) and the peak at lower potential (potential with the highest current density) (Section 3.1). If there were any diffusion barriers, increasing the loading from submonolayer (0.72 μg_Pt_ cm^–2^) to multilayer (10.15 μg_Pt_ cm^–2^) thickness would decrease the catalyst’s specific activities. However, the authors found that the catalyst’s apparent activity remained around 100 mA cm^–2^_Pt_ for ORR and 600 mA cm^–2^_Pt_ for HOR. Thus, they concluded that diffusion barriers did not limit measured activity. A model calculation, with the assumption that the thin Nafion layer behaves like bulk Nafion, showed that the diffusion limitation, which scales with a specific Pt catalyst area, would occur at 3.74 A cm_Pt_^–2^ for the ORR and 1.15 A cm_Pt_^–2^ for the HOR. As mentioned above, the FE-measured ORR current density is at least an order of magnitude lower, and the HOR peak current density is half as low, which means that the ionomer layer is not affecting the measured catalyst activity.^67^ There have been simulations questioning this assumption and suggesting that a denser layer of ionomer forms on the surface of the catalyst.^68^ Thus, while a thin layer of an appropriate Nafion ionomer was not seen to influence the oxygen transport compared to no ionomer,^33^ the next generation of catalysts could benefit from using highly oxygen-permeable ionomers (HOPIs).^68^

**Figure 8:**
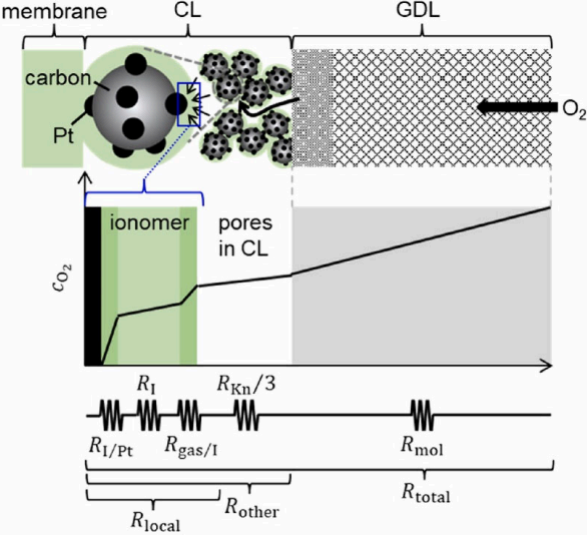
Effects of the gas diffusion layer (GDL), the catalyst layer (CL), and ionomer on the gas transport resistance in the polymer electrolyte membrane electrode and a representative series-circuit model. Reprinted with permission.^69^ Copyright 2023, Elsevier.

To make the FE more closely resemble MEA conditions, the catalyst can be deposited onto a GDL instead of a PCTE substrate. Such bridging methods between RDE and MEA are known as GDEs. The transport of gases will be mainly in the gaseous phase through the GDL and MPL until the catalyst layer (Figure 3). The thickness of these layers can influence the mass transport conditions. The GDE can have a multimicrometer thick catalyst layer, a 50 μm thick MPL supported by a 170 μm GDL (Figure 5).^33,50^ The considerable thickness of the GDL and MPL can play a significant role in mass transport (Figure 8).^69^ In comparison, the PCTE substrate thickness that gas must pass through in the FE is only 12 μm.^33^

Unlike with MEA, the GDE method only investigates one-half-reaction at a time and has a better control of the variables. Compared to the FE, the GDE can use the same materials as will be used in the application later on. This way, optimizing the GDE components–the catalyst layer, GDL, and MPL – one electrode at a time can aid in a more systematic fuel cell or electrolyzer optimization.

In some GDE setups, a membrane can be added between the liquid electrolyte and the catalyst layer.^52^ However, in this configuration, the solid electrolyte (PEM) is always completely hydrated due to contact with a liquid electrolyte in the cell compartment. This contrasts on one side with the MEA, which can have various levels of membrane hydration and on the other side with the FE, which does not have a membrane and is fully hydrated.

A fundamental difference between all other methods described here and the MEA is that in the MEA and in the actual PEMFC system, there is no bulk liquid electrolyte phase. Instead, there is a solid electrolyte, a proton-conducting membrane. Using a more realistic characterization method with a membrane instead of a liquid electrolyte might add necessary information to the guiding principles of catalyst layer design.

By adding a solid electrolyte to the GDE, constant water production from the ORR can flood the electrode, as is also the case with the MEA setup. Due to proton access, high water content can improve proton conductivity and catalyst utilization. Still, at the same time, it can cause the gas channels to flood and close between bulk gas and active sites, which severely hinders gaseous reactant mass transport.^2,51^

The FE setup mitigates intercatalyst layer water accumulation by coating the catalyst agglomerates with a thin layer of hydrophobic Teflon AF.^33^ This makes it possible to assess the maximum possible catalyst activity that could be achieved in a PEMFC.^7,32,33^ However, the ionomer influences the measurement conditions even in the FE, as found by comparing catalyst layers with no ionomer and with different ionomers in the catalyst suspension.^33^ A recent paper using a GDE setup in conjunction with O_2_ transport resistance and CO-displacement measurements confirms that it is vital to balance oxygen mass transport, which decreases with a higher ionomer to carbon (I/C) ratio and proton transport, which increases with a higher I/C.^70^ This is likely the case for all methods that use ionomer in the catalyst layer, which must thus be carefully considered.

### 2.2 *iR* Drop

The fundamental studies aim to measure the potential difference across the electrochemical interface and eliminate, minimize, and compensate for unwanted effects influencing the measurement. In addition to mass transport, another aspect affecting the catalyst activity measurement is the electrolyte and electrode resistance. The *iR* drop is the product of the total current (*i*) and the uncompensated resistance (*R*_u_) between the WE and RE. The *R*_u_ can be measured using electrochemical impedance spectroscopy (EIS), the current interrupt method, small potential steps, or even using the potentiostat positive feedback loop.^12,71^

The ideal electrochemical characterization method for intrinsic catalyst activity determination would have as low an *iR* drop as possible. If *i* and *R* values are known, most of the *iR* compensation (80–90%) would then be done using the potentiostat, which corrects for *iR* drop during the measurement.^12^ Overcompensation can result in instability of the potentiostat (electrode potential oscillations), which can destroy (or “cook”) the electrode due to short but extreme potentials and current densities.^12^ Thus, some of the compensation (10–20%) should be done during data analysis. If all of the compensation is done after the measurement, instead of dynamic or positive compensation, then oscillation is not a problem, but the scan rate will no longer be constant. Scan rate affects the electrode state (e.g., coverage of Pt electrode with oxide layer) and mass transport conditions.^12^

In order to reduce the required correction, and hence avoid the possibility of introducing errors, it is desirable to operate at low currents. This also means that the primary potential distribution at the electrode/electrolyte interface is as uniform as possible,^72^ leading to uniform current distribution. Low *iR* drop limits local heating effects due to Joule heating. Low *i* in *iR* drop can be achieved by reducing the electrode area and the catalyst loading to a level where the total current would be as low as possible, yet measurable, with the available potentiostat while retaining the accuracy of current measurement and ensuring reproducible electrode preparation. In addition to reducing the *i* component of the *iR* drop, small electrode areas and thin catalyst layers prevent a phenomenon where there is a significant difference in overpotential for different catalyst particles far apart from each other laterally or longitudinally in the catalyst layer. Experimentally, the inhomogeneity of the experienced overpotential for large and thick electrodes can cause the reduction of the resolution of fine structure in the voltammetry measurement, as exemplified in Section 3 of this review, where the fine structure of the *iE* curves in the FE and GDE measurements is compared.

The *R*_u_ can be minimized by a highly conductive electrolyte and by positioning the Luggin capillary close to the WE, where the short distance between the electrode and the Luggin tip means that the *iR* drop is smaller. However, the Luggin capillary must not be positioned so close that shielding effects are introduced. The shielding effects occur when something near the electrode, such as a Luggin capillary, causes a change in the primary potential distribution near the working electrode coincident with the Luggin capillary and a partial blockage of the mass transport path in the solution.^73^ The optimal distance and position with regard to the WE depends on the method used and the aims of the experiment.^74,75^

With the RDE technique, the tip of the Luggin capillary is positioned next to the RDE on the same plane as the RDE tip to reduce shielding effects disrupting the electrolyte flow directly under the RDE tip. For methods such as the FE and GDE, the Luggin capillary can be positioned closer to the electrode in the solution phase because the main mass transport of reactants occurs on the other side of the electrode in the gas phase. A reference electrode can also contact the working electrode using the membrane.^76^ This might, however, require a more rigorous analysis, which should not be ignored.^77^

Electrolyte conductivity can be increased by higher temperatures, but not without complications, including faster electrolyte evaporation and the resulting change in electrolyte concentration. Higher electrolyte concentration also increases its conductivity, but this comes at the cost of higher ionic strength, which can have adverse effects. A 30–40% decrease in ORR specific activity was observed when increasing the HClO_4_ electrolyte concentration from 0.1 to 1 M in an RDE experiment.^7^

The necessity for *iR* drop correction increases with increasing *i*, with UME not needing any compensation and the significance increasing in order of methods RDE, FE, MEA, and GDE, as exemplified in Figure 1 and noted in Table 1 below.

However, most MEA measurements are done without compensation because the aim is to compare the results to a real system where potential compensation will not occur either. The same can be argued for some GDE experiments that aim to replicate the MEA conditions.

### 2.3 Other Variables

Aside from measuring the relationship between potential and current, controlling other variables can increase the reliability of the measurements and give extra information about the reaction mechanisms on the catalyst. Such variables include the temperature, pressure, humidity, pH of the electrolyte, catalyst loading, and the electrode area (Table 1).

#### 2.3.1 Temperature and Pressure

The rate of reactions in electrochemical processes is highly dependent on temperature—an increase in temperature results in faster reaction kinetics. Additionally, temperature changes can affect the electrolyte solution’s conductivity and viscosity. All of which can influence the measurements. Pressure can also influence the equilibrium concentration in the solution and the equilibrium potential of the reaction being measured.

Although some unique configurations have been proposed, the typical upper limit of temperature and pressure of UME, RDE, and FE is usually about 60 °C and 1 bar.^36,59,78,79^ The main issues involve electrolyte evaporation and precise control of temperature. For some GDE setups, higher temperatures and pressures are possible, and fuel cell-relevant conditions can be achieved.^76,80^ However, deconvoluting the effects of temperature, pressure, and humidity on GDL, MPL, and the catalyst layer (ionomer and catalyst) is challenging in GDE and MEA setups.

All in all, to investigate the intrinsic activity of a catalyst, it is essential to carefully control and monitor the temperature and pressure during electrochemical characterization to ensure accurate and reliable results. Once the intrinsic activity is known, GDE and MEA should be used to optimize the operating conditions for the developed catalyst.

#### 2.3.2 Humidity

In UME and RDE, the electrode is submerged in an electrolyte, which means the catalyst layer is flooded and has good conductivity. Changing the humidity conditions in UME and RDE is impossible. In the case of the FE and GDE, assuming that the relative humidity is in equilibrium with the water pressure over the acid, the proximity of the gas-accessible catalyst layer to the electrolyte causes the relative humidity to change very little from close to 100%. However, the humidification of the gases can start to play a role with thicker catalyst layers of GDE.^45^

High humidity can cause water management problems for fuel cells.^7^ Thus, as the actual application of the developed catalysts can have less than 100% relative humidity,^37^ it is vital to measure the activity response to lower humidity levels later in the PEMFC catalyst development process. Therefore, optimizing the humidity conditions using the MEA method without a liquid electrolyte is necessary before application.

#### 2.3.3 pH

The pH can impact the rate and mechanism of the reaction. Whether a method can be used to investigate reactions in environments with different pH depends mainly on the materials used in the setup. While glass cells are suitable for strong acid electrolytes, in bases, impurities can be leached out from the glass into the electrolyte, which affects electrochemistry. However, fluoropolymers such as polytetrafluoroethylene (PTFE) cells, which resist concentrated strong bases and acids, can be used. On the other hand, PTFE cells are not transparent, which means that the setup and visual monitoring of gas evolution and other processes is complicated.

The FE uses a porous PCTE membrane as its WE substrate. Although polycarbonate is stable in low pH, its resistance to high pH is poor. This limits the FE applicability to acidic or only slightly alkaline environments until a suitable alternative substrate is found. GDE methods, on the other hand, have been used with both acidic and alkaline electrolytes because they can use the same materials as the respective fuel cells. Although it does not mean there cannot be a degradation of the components at extreme pH, the performance measurements will resemble the real systems, where the same processes can, in such cases, occur as well.

High current densities and high catalyst loadings can make the pH effects even more problematic due to the local production or consumption of protons, which could lead to a marked change in local pH. Additionally, the difference in proton concentration can cause a significant unaccounted geometric flux of the electrolyte due to the movement of protons or hydroxide ions.

At the intermediate pH, buffer solutions are required to counteract significant pH changes in the electrolyte solution at the electrode if high current density methods are used.^81^ The selection of such buffers must be carefully considered, as many can interact with the electrocatalysts.^82^

pH control is important in all methods with a liquid electrolyte. On the other hand, due to the absence of a liquid electrolyte in real fuel cells, the discussion is more focused on potential, current density, temperature, and pressure effects on performance, as these are the main levers of control. However, some efforts have been made toward a direct pH measurement in the MEA.^83^ This can become more valuable in the upcoming years as alternatives to fluoropolymers, such as Nafion, are investigated.

#### 2.3.4 Catalyst Loading

The deposition method and related benefits and downsides depend on the electrode size and loading. While UMEC, FE and RDE have relatively small electrode areas, GDE method electrode sizes can range from 0.07 to 2 cm^2^, which start to resemble the MEA electrode sizes^8^ (Table 1). While MEAs in real fuel cells are typically several hundred square centimeters in geometric area, the test systems may be as small as 4 cm^2^. There are inherent benefits to using both large and small electrodes and loadings. Electrode’s large size and high loadings can improve catalyst layer homogeneity and make the true catalyst loading more accurate and easily determinable. Additionally, large laboratory electrodes, such as the spray-coated 2.01 cm^2^ electrode by Ehelebe et al.,^8,9^ can use the same manufacturing methods as applied for commercial fuel cells and electrolyzers. Using the same coating method makes the GDE performance more comparable to those of commercial cells.^8^ This means coating optimization could also be done on GDEs one electrode at a time rather than using the more complicated MEAs. The effect of different conditions in GDE and MEA outlined in previous sections should still be considered.

With large electrodes and high loadings, the local potentials and reactant concentration can differ at different locations and through the thickness of the electrodes, leading to nonuniform conditions at the catalyst surface. This could be why the *iE* curve’s fine structure in the activity measurements has not been reported using the GDE methods. Additionally, a larger geometric current caused by a higher loading leads to more significant *iR* drops, local heating effects and the possibility of reactant starvation, which can result in mass transport effects.

GDEs are designed to mimic MEAs, and therefore, the typical 100 μg_Pt_ cm^–2^ loading spray-coated onto their substrate is equal to MEA loadings. However, reducing the loading is possible. For MEA, the most recent methods have tested a uniform 5 μg_Pt_ cm^–2^ layer on each side, totalling 10 μg_Pt_ cm^–2^, comparable to a standard RDE measurement.^7^ The VFC technique for GDE and MEA could also create more uniform, low-loading catalyst layers.^22,33,42^ The question remains whether it is desirable to investigate low-loading GDE and MEA when the real PEMFCs use 100 μg_Pt_ cm^–2^ loadings and above. To avoid losses in the spray-coating setup tubes and nozzle, which can add up to even a gram of catalyst, vacuum deposition was tested for MEA catalyst layer manufacturing, where the researchers deposited the usual 100 μg_Pt_ cm^–2^ loading onto a GDE but only used 30 mg of catalyst material.^84^ Recently, inspired by the FE technique, ultralow loading (5.2–7.1 μg_Pt_ cm^–2^) catalyst-coated membranes were produced using the PCTE and VFC.^85^

Low loadings are better for determining the catalyst’s intrinsic activity as the mass transport within the catalyst layer has a lesser effect. Additionally, the need for a smaller amount of catalyst can be useful in early stage catalyst research. Theoretically, the UMEC setup requires the smallest total amount of catalyst (0.0075 μg_Pt_) per experiment,^14^ but in reality, more must be used during the filling process. It must be noted, however, that the cavity has a height of 29 μm, which makes the catalyst layer an order of magnitude thicker than a TF-RDE catalyst layer (approximately 2 μm).^14^ RDE catalyst layers are usually made using drop casting. This process has been extensively optimized to provide uniform layers for loadings down to 4 μg_Pt_ cm^–2^.^86^ More commonly, the RDE experiments are done using a loading of around 10–20 μg_Pt_ cm^–2^ to ensure layer uniformity.

The most recent application of the FE used about 0.5 μg_Pt_ cm^–2^ of 10 wt % Pt catalyst.^87^ Typically, 1 μg_Pt_ cm^–2^ or higher loadings are used with a 2 mm diameter catalyst spot size. The FE method uses a flat gold-coated PCTE substrate with precisely formed pores, allowing for uniform catalyst coating even at the smallest loadings using the self-leveling VFC deposition technique.^32^ It must be kept in mind that the equilibrium coverage of contaminants on the catalyst surface is obtained fastest with small amounts of catalyst and electrolyte (and contaminant) convection. Thus, the cleanliness levels should be as high for the FE with ultralow catalyst loadings as for the RDE with electrolyte convection.

Low loading makes reproducing electrodes and determining the precise loading much more difficult, as even a tiny deviation in loading can result in a significant change in current density measurement.^87^ To determine the actual loading of the catalyst on the low-loading technique’s electrode, the electrode can be digested in *aqua regia*, and the metal concentrations measured using the inductively coupled plasma mass spectrometry (ICP-MS).^34^ Alternatively, electrochemically active surface area (ECSA) values can be used to back-calculate the catalyst loading if the actual ECSA values of the catalyst are known.^32^ Most commonly, the ECSA is determined by the hydrogen underpotential deposition (H_UPD_) or CO-stripping method. It has even been suggested to consider copper underpotential deposition (Cu_upd_) for ECSA analysis and loading determination in some cases when alloys such as PtRu are used.^88,89^

Early stage characterization with very low amounts of catalysts is generally more straightforward with UME, RDE, and FE methods. Still, with new advanced coating methods, the catalyst loading could be reduced so that any preferred method can be used. However, later-stage characterization of layers with higher loadings would be preferable to give valuable insights into application-focused catalyst layer characteristics.

### 2.4 Cleanliness

Different cleanliness standards should be applied depending on the experiment’s goal. The intrinsic activity and mechanism investigations require much lower levels of impurities than methods assessing the realistic activities that use GDLs and membranes.

The lower the catalyst loading, the more problematic the impurities are, as the critical coverage of contaminants is achieved faster, even with low levels of contaminants.^32^ This means the standard is to clean the glass or fluoropolymer cells using highly oxidizing solutions (e.g., a mixture of concentrated sulfuric acid and hydrogen peroxide). Optimal protocols for cleaning and testing the cleanliness of the cells are available for RDE^22^ and FE.^33^

Even after cleaning the cell, impurities from the electrode can remain in the catalyst layer due to the components like isopropanol, Nafion and the catalyst itself. As the electrode cannot be washed with an acid, complete removal of impurities is difficult. Drying the electrode in a vacuum oven can eliminate most of the solvent and other volatile organic matter, but some likely remain. Oxidizing the remaining organic impurities is one of the aims of the break-in procedure.^22,33^ Such pretreatment protocols have been developed for RDE,^22^ FE,^33^ GDE,^42^ and MEA^37^ methods. However, the break-in cleaning effect can be short-lived if contaminants are desorbed from the catalyst surface to the bulk electrolyte and later readsorb. This is especially problematic for low-loading and hydrodynamic methods, where electrochemical cleaning must be done throughout the experiment to keep the electrode surface clean.

Clean high-concentration acids, such as double-distilled acids, are necessary to keep the levels of impurities down in the electrolyte when high-concentration electrolytes are used to improve conductivity and reduce the ohmic potential drop. On the other hand, double-distilled acids are expensive, and high concentrations of clean acids still cause higher anion, such as sulfate, adsorption on the catalysts, which decreases the catalyst activity.^90^ Therefore, it is recommended that cleanliness, conductivity, and anion adsorption be considered before the experiment. In the FE experiments, where about 100 mL of electrolyte is used, the initial 4 M HClO_4_ electrolytes^32,62^ were replaced with 1 M HClO_4_ electrolytes in later reports.^33,34^

### 2.5 Reporting Catalyst Activities

Activity can be reported with regard to the ECSA, catalyst mass, or electrode geometric area (Table 2). Focusing on increasing the geometric activity is an essential aspect of fuel cell production when the catalyst material has already been optimized. However, as the geometric activity can usually be increased by having more catalyst material, it is of little use in the search for better catalysts. Reporting the activity as specific activity (SA) is the most appropriate when the experiments have focused on tuning the catalyst composition or surface structure—from monocrystal to geometrically complex alloys. The turnover frequency is a closely related alternative to SA, but it could cause some confusion because different units are used.^91^ The central idea is to relate the physical characterization of the catalyst surface to the electrochemically measured catalyst activity.

**Table 2:** Aspects to Consider When Choosing a Type of Activity Reporting

Type of Activity	Aspects to Consider
Specific Activity (A cm^–2^_Pt_) or Turnover Frequency (electron site^–1^ s^–1^)	• It is the intrinsic catalytic activity of the catalyst material.
	• Focus on catalyst composition and surface structure.
	• Accurate determination of ECSA is the priority.
	• Accurate loading determination is less critical.
	
Mass Activity (A g^–1^_Pt_)	• It is the ability to produce high ECSA per unit of mass of catalyst material while retaining the specific activity.
	• Focus on increasing the specific surface area with the same composition structure, e.g. by increasing the dispersion of the catalyst.
	• Directly relates to the cost of the required catalyst to produce a given performance.
	• Accurate determination of loading is the priority.
	
Geometric Activity (A cm^–2^_Geo_)	• It is the total performance of the produced electrode.
	• Focus on determining the optimal catalyst loading of the electrode.
	• Does not aid as much in the search for better catalysts.
	• Relates to the total area of the electrode and the optimal loading needed to produce a given power output.
	• Accurate determination and uniformity of loading are the priorities.

Mass activity (MA) is associated with the ability to produce as many of the developed active catalyst sites per unit of catalyst mass as possible. This is a crucial factor in commercialization, where the mass of the catalyst determines the cost. While the reduction in particle size is the easiest way to increase the MA, a platinum catalyst can exhibit the so-called “particle size effect” when the SA decreases with a decrease in particle size.^92^

Based on that, we outline a generalized technical approach from catalyst synthesis to commercialization:

a) Maximization of the SA of the catalyst at the application-relevant potentials by optimizing the composition and surface structure using the FE.
b) Maximization of the MA by increasing the specific surface area with the same composition and surface structure using the GDE.
c) Optimization of the catalyst loading to produce the required geometric activity using the MEA.

It is important to note that the way the activity is measured and calculated also plays a significant role in the comparability of the results. If the activities are reported as specific activities but one experiment is done with cyclic voltammetry with 20 mV s^–1^ and the other with 200 mV s^–1^, then a direct comparison of the results must be done very carefully.^93^ Even more difficult is to compare results from different types of activity measurements, such as cyclic voltammetry and galvanostatic steps.^8^ Even small aspects, such as the potential range and scan direction, can influence the activity due to different surface conditions of the catalyst.^22^ This can be visible from the measurements as hysteresis.^22^

SA calculation requires the knowledge of ECSA. Because H_UPD_ and CO-stripping ECSA values can differ,^33^ the SA can also change accordingly. The reason for differences in ECSA values could be varying adsorption strength of species such as hydrogen, CO, and electrolyte or ionomer anions. A philosophical question arises as to whether it is appropriate to use the available ECSA (measured with H_UPD_) or total ECSA (measured with CO-stripping), which includes a degree of blocked sites for specific activity calculation. It is an open question of how to measure the available active catalyst surface area as a function of potential. Commonly, this can only be estimated through activity measurements.^35,62^ To summarize, it is vital to meticulously report and pay attention to the experiment conditions when comparing electrochemical measurements.

## 3 Catalyst Intrinsic Activity Findings from FE Experiments

### 3.1 Platinum *iE* Curve Fine Structure

**Figure 9:**
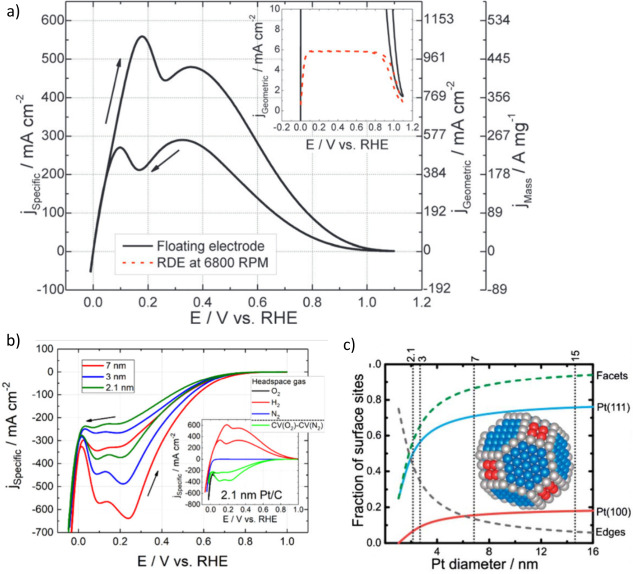
(a) A comparison of the floating electrode and the rotating disc electrode (RDE) for measuring hydrogen oxidation reaction activity with *iE* fine structure visible between 0.1 and 0.5 V vs RHE. Reprinted with permission.^32^ Copyright 2013, Royal Society of Chemistry. (b) The oxygen reduction reaction-specific activity on different size Pt/C nanoparticle catalysts as measured by the floating electrode with *iE* fine structure visible between 0.05 and 0.3 V vs RHE. Reprinted with permission.^62^ Copyright 2020, American Chemical Society. (c) A schematic of a truncated octahedron platinum particle and the fraction of different facets to surface sites with regards to particle size. Reproduced under the terms of the Creative Commons Attribution 3.0 Unported (CC BY 3.0) license. Copyright 2017, Royal Society of Chemistry.^94^

A good resolution fine structure in *iE* curves can give valuable insight into the reaction mechanism.^32^ The HOR fine structure has been a topic of discussion for many articles, which have suggested different reasons for it.^32^ Further research on the fine structure could lead to better understanding and design principles for catalysts. In the FE experiments, thanks to very low loadings (0.5–2 μg_Pt_ cm^–2^) and low catalyst layer resistance, a good resolution of the ORR *iE* curve fine structure, similar to what was found earlier in HOR investigations, was present in the ORR high overpotential region (Figure 9).^32^ The *iE* curve peaks were assigned to facets and edges of the Pt particles, with facets dominating the ORR at about 0.24 V and edges at about 0.1 V vs RHE. The low coordination sites are likely only active toward ORR at low potentials and drop off at above 0.3 V vs RHE due to the adsorption of oxides or anions, which block the catalyst surface and thus decrease the activity.^32^ Additionally, increasing the size of the nanoparticles increases the ratio of facets to edges, corresponding to a truncated octahedron geometry, which changes the ratio of the *iE* curve peak heights. Below the potential of this edge site’s active peak at 0.1 V vs RHE, the ORR rapidly decreases due to hydrogen adsorption on the catalyst, blocking the sites for ORR but remaining active toward HER.^62,94^

### 3.2 Intrinsic Oxygen Reduction Reaction Activity and Blocked Surfaces

**Figure 10:**
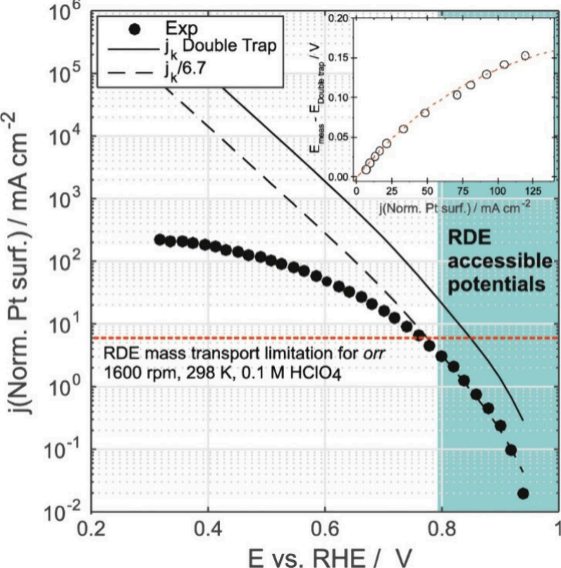
Experimental results of the floating electrode for the oxygen reduction reaction (ORR) by Markiewicz et al.^35^ are compared to the ORR kinetic current density calculated using the double trap model by Wang et al.^60^ (solid line) and the scaled double trap model by Wang et al.^97^ (dashed line). The potential region where the rotating disk electrode (RDE) can adequately assess the ORR kinetics is shaded. Inset compares the difference between the FE experimental data and the scaled double trap model as a function of specific activity. Reproduced under the terms of the Creative Commons Attribution 4.0 International (CC BY 4.0) license. Copyright 2015, Elsevier.^35^

Models, such as the Koutecký-Levich model^95,96^ or the double trap model by Wang et al.,^60^ are used to calculate the kinetic activity of catalysts from RDE measurements in the mixed kinetics region.^12^ Therefore, the intrinsic ORR activity of Pt-based catalysts can be estimated by modeling the RDE experimental data. Efforts have been made to include fewer assumptions in the kinetic models, for example, without assuming which step is rate-determining like was done when developing the double trap model.^60^ However, as was observed by Markiewicz et al.,^35^ the kinetic currents calculated with a double trap model, even after scaling, *as per* Wang et al.,^97^ deviate from high mass transport FE ORR experimental data at RDE-inaccessible potential range (Figure 10). Markiewicz et al.^35^ then modified the double trap model by including the formation of OOH_ad_ intermediates, resulting in a model that shows good agreement with FE experimental data even at large overpotentials. Additionally, the model includes the fewest parameters possible to reduce overparameterization.^35^

From the modified double trap model, the coverage of different adsorbates in a wide potential range can be derived, which can then be used to calculate the free site proportion. The FE was used to compare the influence of different sizes of Pt and PtCo particles on ORR.^62^ Then, the modified double trap model was used with the addition of considering two different catalytic sites.^62^ In addition to facets regarded as the central active sites in the previous model, edge sides were now included due to their unique activity at very low potentials (Figure 9c). The model by Markiewicz et al.^35^ was upgraded to include the changing oxygen binding energies of Pt nanoparticles, which can occur with changing ratios of edge and facet sites or catalyst composition. Applying the developed model to the measured data, it was found that about half of the PtCo catalyst was blocked by adsorbates. Thus, if the adsorbate-free surface area of PtCo catalysts could be increased, the activity could be doubled. Zalitis et al.^62^ concluded that the measurements in the operational potential range (0.62–0.76 V vs RHE^62^) cannot be reliably replaced with extrapolation as the adsorption strength and coverage of the adsorbates and intermediates can change according to appropriate adsorption isotherms.^12^

### 3.3 MEA vs GDE vs FE

While the full potential range can be investigated in the MEA measurements, the complexity of the setup makes the analysis of the intrinsic catalyst activity very difficult, if not impossible. In MEA studies, the ORR activity measured by RDE for the commercial catalyst is rarely achieved. Furthermore, the gap widens with novel catalysts.^98^ One reason for the gap in activity measurements between RDE and MEA setups could be due to oxide coverage on the catalyst surface.^7^ It might be that the novel catalyst is not as significantly more active toward ORR but that less of the surface is deactivated with oxides at RDE measurement potentials. At FC-relevant potentials, the new catalyst might underperform because the baseline catalyst is also oxide-free. This can be seen from an FE measurement comparison between Pt and PtCo ORR catalysts, where the activity gap of the two catalysts decreases at low potentials.^62^ The only way to differentiate between the effects of oxide coverage and the intrinsic activity of a catalyst is to measure its activity at a wide range of potentials and use appropriate models to estimate the available ECSA.^35^ The ability to make very low-loading MEAs may make it easier to investigate intrinsic catalyst activities,^85^ although there are still some confounding issues, such as hydrogen crossover affecting the performance of very low-loading systems.

**Figure 11:**
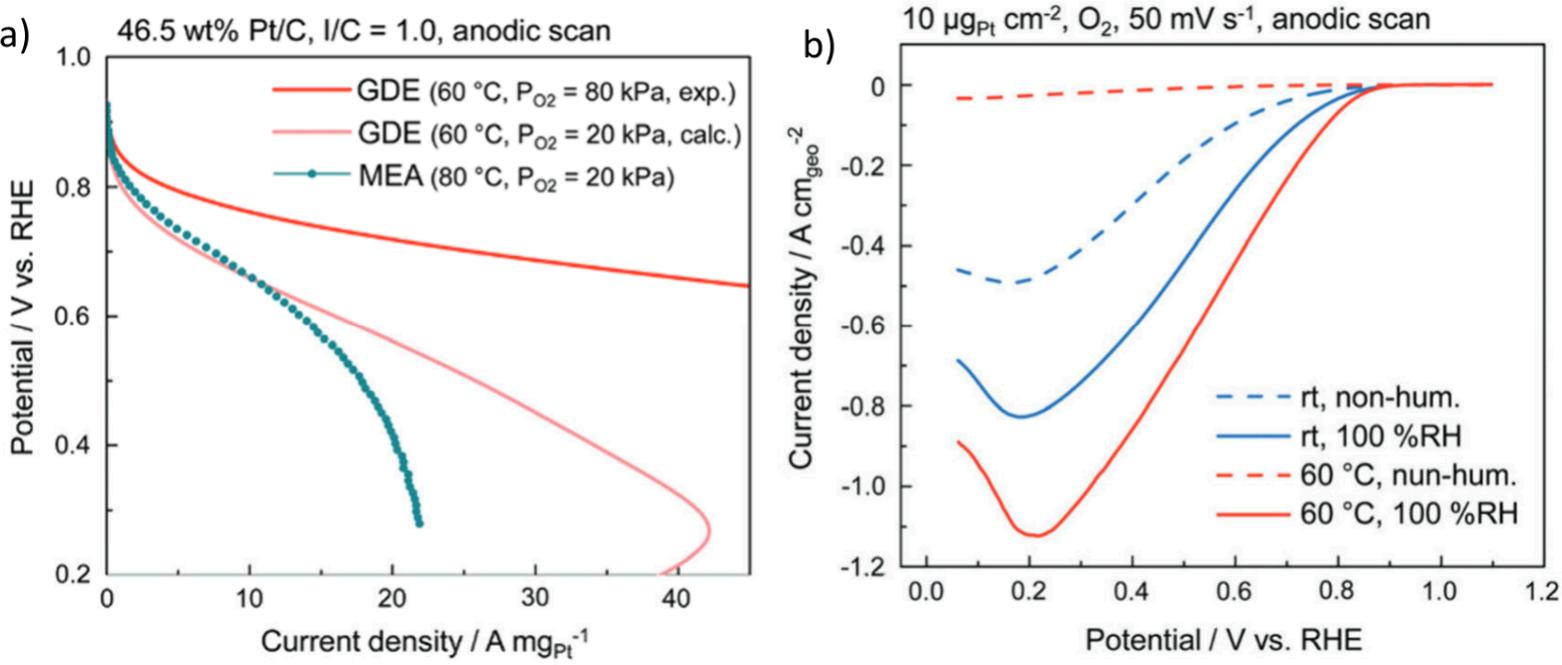
(a) Gas diffusion electrode (GDE) oxygen reduction reaction activity measurements compared to membrane electrode assembly (MEA) results, and (b) GDE activity comparison at different conditions. Reproduced with permission.^42^ Copyright 2008, Royal Society of Chemistry.

GDE can replicate the MEA results well but with lower catalyst amounts and better control of the variables (Figure 11). This means that conditions can be tuned to achieve better performances compared to MEA. Solid electrolyte effects can be more clearly investigated because GDEs work with and without a membrane. All these aspects make GDEs valuable tools to use before MEA optimization. However, while achieving comparable specific current densities to MEA measurements, the results achieved with the GDE setup have not shown the two separate peaks in the ORR *iE* curve fine structure area (0.05–0.30 V vs RHE) seen in the FE experiments (Figure 11b).

**Figure 12:**
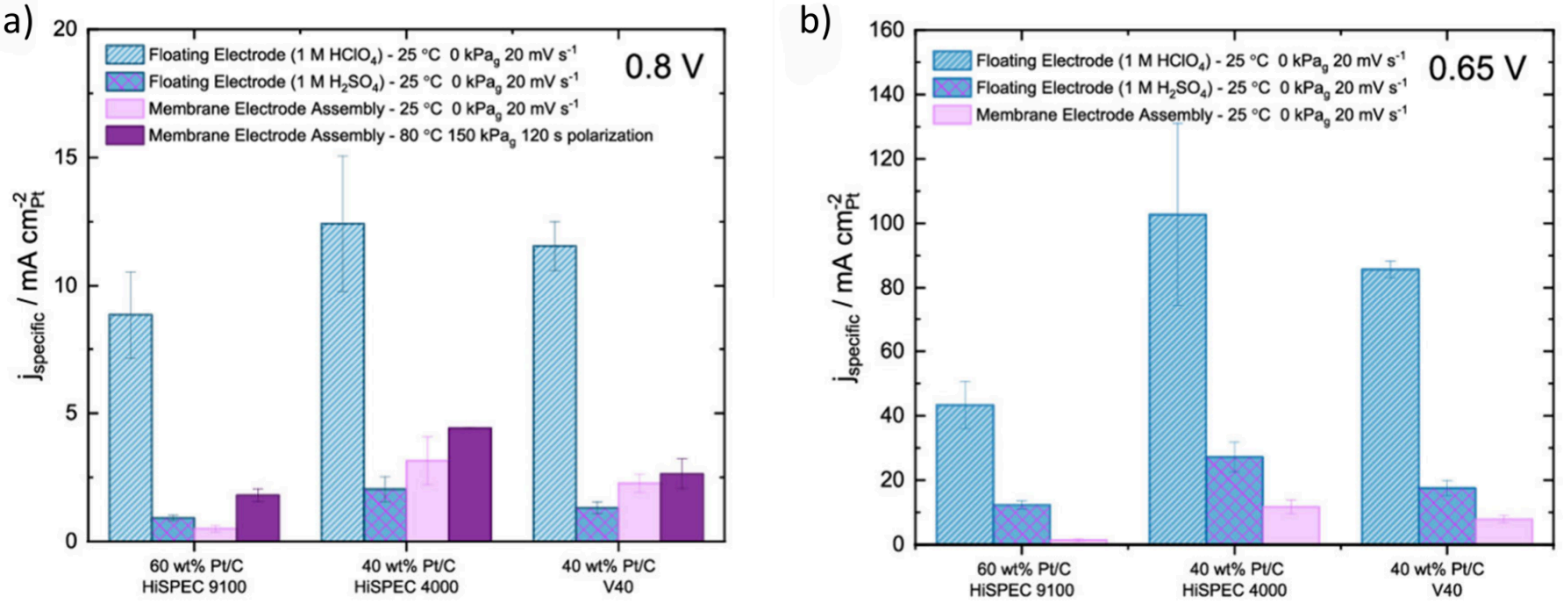
Oxygen reduction reaction activity comparison with the floating electrode and membrane electrode assembly at 0.80 V (a) and 0.65 V (b) vs RHE. Adapted with permission.^7^ Copyright 2022, American Chemical Society.

Jackson et al.^7^ compared three commercial platinum catalysts using FE and MEA techniques with loadings of 2.4–3.8 μg_Pt_ cm^–2^ and 400 μg_Pt_ cm^–2^, respectively (Figure 12). They showed that the activity of commercial catalysts measured with FE at room temperature and pressure is higher than what MEAs achieve at 80 °C and 150 kPa. They envisaged that current densities up to 16 A cm^–2^ at 0.65 V vs RHE could be achieved when the optimization improves for the MEAs with a catalyst loading of 400 μg_Pt_ cm^–2^.^7^ Most importantly, there is not a clear rate-determining step and both catalyst activity and MEA optimization contribute to the overall performance of the fuel cell. This means the FE can be used to develop catalyst materials with higher intrinsic activities and to set a performance target at the fuel cell’s relevant potential range. Then, GDEs and MEAs can be used to optimize the fuel cell layers and conditions to achieve the set target.

## 4 Wider Scope and Future Prospects

Although the applications of the FE and different GDE methods have been discussed,^99^ and some articles have briefly included one or the other in their experiments, a systematic experimental study should be done analyzing the comparability of results from the different methods. This could give a clearer understanding of where each method excels and pave the way for future improvements in FE and GDE optimization or aid in developing the next generation of electrochemical characterization methods. Such experiments could include replicating the comparison of oxygen mass transport in nitrogen and helium in the different GDE setups^32^ and validating how well the GDE and MEA optimization results, e.g. catalyst layer composition and deposition method, match. For FE, interlaboratory testing, including the experimental protocols outlined by Ehelebe et al.,^8^ should be done and measured catalyst activities compared to the GDE methods.

In addition to ORR, FE and GDE have been used to investigate the HOR,^34,79,94^ HER,^79,94^ OER,^49,100−103^ CO reduction,^104−106^ CO_2_ reduction,^102,107−111^ methanol and ethanol oxidation reactions,^80^ and even to study reaction kinetics in metal-air-batteries.^112^ Each of these reactions either uses a gaseous reactant or produces gases during the reaction, and thus, good mass transport conditions for gases can be beneficial for the investigations. FE and GDE methods should be used more extensively to gain valuable insights into these reaction mechanisms that cannot be obtained with the RDE or RRDE methods. Reducing nitrogen oxides (N_2_O, NO and NO_2_)—greenhouse gases^113^ and pollutants^114^—or even the conversion of nitrogen gas to ammonia (NH_3_) can also be an avenue for research with the FE and GDE methods.

As the potential for better design principles for non-PGM catalysts is even higher than for PGM catalysts, their development using the new FE and GDE methods should also be a priority. This has been done for some GDEs,^115^ but FEs are currently limited to systems with a pH below 10. An alkaline-resistant FE setup should be built to increase the pH values where it can be used. An alkaline FE could be used to investigate non-PGM catalysts such as peat-derived carbon doped with iron for anion exchange membrane ORR catalysts.^116^ Another exciting avenue for gas-accessible electrochemical characterization methods could be to use alternative electrolytes like aprotic organic electrolytes.^117^ Possibilities also include using organic solvents (for direct ethanol fuel cell catalyst development) or ionic liquids (for carbon dioxide reduction reaction^118^).

EIS, a powerful tool that uses alternative current perturbations to get information about impedance at different frequencies, should also be used more extensively in FE and GDE setups.^119,120^ EIS can give valuable information about the reaction mechanism, local reaction conditions and differences between the methods. The GDE experiments can and should already be used to examine the impact of GDLs and membranes and to aid in optimizing variables before MEA testing. Additionally, accelerated stress tests (ASTs) in GDEs could be valuable alternatives to such tests in the MEAs,^44,121^ but their limitations should be kept in mind.^122^ For example, drawing conclusions from degradation experiments might be difficult as the liquid electrolyte can flood the GDL of the GDE, which is not an issue in an MEA.^103^ The FE has no liquid ingress with time, but the materials used in the FE and MEA are quite different. Thus, further research with MEA, GDE, and FE should be done to assess how the GDE and FE methods should be used for degradation tests and what benefits they bring over using an MEA setup in the first place.

If membranes could be incorporated into the FE setup, it could also be used to investigate the membrane effects on the reaction kinetics. Eventually, when the FE is used more extensively to measure the intrinsic activity most closely resembling mass-transport-free conditions, that data can be the base for creating more accurate computational models. As the apparent activity is influenced by components in the system, such as the GDL, MPL, and membrane, developing kinetic models based on the GDE experiments is more complex. However, machine learning algorithms could potentially be used for that application without the need to understand or separate the effects of the components from the resulting activity.

Future research should also make use of the various tandem methods developed for both FE and GDE in areas such as photoelectrochemistry^123,124^ and spectroelectrochemistry.^102,125^ While significant modifications must be made to the setups if *in situ* NMR^126^ or EPR^127^ spectroscopy is of interest, other tandem methods have already been reported. The gas-accessible membrane electrode (GAME) developed by Zhang et al.^109^ used a modified FE and a UME to achieve RRDE-like product probing capabilities and integrated an online electrochemical mass spectrometer (OLEMS) to measure the carbon dioxide electrochemical reduction reaction product gases with less than a seven-second delay.^109,110^ They also suggested incorporating a Fourier-transform infrared (FTIR) or Raman spectrometry to probe the changes on the electrode surface *in situ* during the electrochemical experiment.^109^ In the future, FE with UME could be a helpful tool to investigate HER, which happens as an undesirable side-reaction during the electroplating of electroactive metals like zinc or cobalt.^128^

A GDE method by Hrnjić et al.^48^ used a catalyst-coated transmission electron microscope (TEM) gold grid with a GDL on top. This means the catalyst can be investigated at the same location before and after the experiment in an identical-location TEM (IL-TEM) “spot the difference” game, used previously with a modified RDE setup.^129^ The IL-TEM method was helpful for the analysis of processes during degradation.^130,131^ Incorporating IL-TEM has been suggested for other GDE methods as well.^44^ Degradation was also studied with small-angle X-ray scattering (SAXS)^132^ and Bele et al.^49^ added the possibility of identical location scanning electron microscopy (IL-SEM), X-ray photoelectron spectroscopy, and Raman spectroscopy. On the other hand, Sherwin et al.^102^ and Gebhard et al.^133^ developed a setup for in-operando synchrotron X-ray and neutron imaging. Most GDE methods could also use *in situ* gas exhaust characterization methods such as differential electrochemical mass spectrometry (DEMS)^9,117^ or conduct electrolyte characterization with ICP-MS.^47,121^ A special GDE was built to perform membrane-less flow electrolyzer mass spectrometry (FEMS).^106^ However, it must be kept in mind that the incorporated characterization methods are only reliable for the specific measurement conditions, and extrapolation to other conditions must be done carefully. In summary, the FE and GDE can and have been easily paired with analytical techniques to investigate the gas phase, electrolyte, and catalyst before, during, and after the experiment.

## 5 Conclusion

PEMFC ORR catalysts’ activity and durability must be improved for broader commercialization. Doing so systematically requires a better understanding of the reaction mechanism on the catalyst. The developed catalysts should then undergo streamlined screening and optimization with increasing granularity.

Each characterization method has unique benefits, downsides, and a place in the catalyst research. UME is best for fundamental studies of defined metallic surfaces. While UMEC can investigate catalyst powders, the thick catalyst layer has non-uniform catalyst utilization, thus underestimating the catalyst activity. The RDE is the most used and understood method, and its simplicity makes it the best screening tool for electrochemical catalysts. The RRDE is similarly accessible and standardized with the addition of being able to measure some reaction products, which can lead to mechanistic insight. However, both can only be used to characterize catalysts at low overpotentials, and extrapolation of reaction rates (and mechanism) to mass transport limited current densities must be avoided.

The FE, with its unparalleled mass transport conditions, can give a unique insight into the intrinsic activity of the catalysts at a wide range of potentials and current densities and shed light on ORR and HER mechanisms in high mass transport conditions, which cannot be achieved with other methods, paving the way to designing better PEMFC catalysts. Additionally, the adsorbate coverage model can provide valuable information about the catalyst surface. However, aside from the high mass transport, the carefully designed FE measurement conditions do not mirror those in the fuel cell, where the effects of the catalyst layer, membrane, GDL, and higher temperatures and pressures make achieving the intrinsic activity of the catalyst unlikely. On the other hand, incorporating the membrane and GDL, such as in the GDE and MEA methods, will mask the intrinsic activity with the effects of different components. Nevertheless, the GDE is a remarkable bridging tool that can streamline the catalyst commercialization from the development level to the stack level by creating PEMFC-like conditions with less effort than a conventional MEA. Thus, by having reasonable control of components and conditions, their effects on MEA performance could be investigated. The possibility of using a GDE with and without a membrane makes it a unique tool for assessing the impact of membranes on novel catalysts. Ultimately, the MEA is still an irreplaceable tool for characterizing catalysts in actual working conditions. Regardless of what the previous characterization methods claim, the results in the MEA are the closest to the performance achieved in the application.

Thus, incorporating a range of characterization methods into the electrocatalyst development process is encouraged. The availability of materials and instructions means that the entry bar for these advanced methods is low. When the high mass transport methods become commonplace, more correct models of reactions will follow, using kinetic data not hidden in the mass transport resistances or extrapolated using unrealistic assumptions. However, this will only be the case if both possibilities and limitations of the methods are understood. If properly applied, these new methods and models will pave the way towards development of catalyst materials that quickly make the transition into real devices.

## References

[ref1] Wang Y.; Pang Y.; Xu H.; Martinez A.; Chen K. S. PEM Fuel Cell and Electrolysis Cell Technologies and Hydrogen Infrastructure Development – a Review. Energy Environ. Sci. 2022, 15 (6), 2288–2328. 10.1039/D2EE00790H.

[ref2] Mo S.; Du L.; Huang Z.; Chen J.; Zhou Y.; Wu P.; Meng L.; Wang N.; Xing L.; Zhao M.; Yang Y.; Tang J.; Zou Y.; Ye S. Recent Advances on PEM Fuel Cells: From Key Materials to Membrane Electrode Assembly. Electrochem. Energy Rev. 2023, 6 (1), 28 10.1007/s41918-023-00190-w.

[ref3] Gröger O.; Gasteiger H. A.; Suchsland J.-P. Review—Electromobility: Batteries or Fuel Cells?. J. Electrochem. Soc. 2015, 162 (14), A2605–A2622. 10.1149/2.0211514jes.

[ref4] Shiva Kumar S.; Himabindu V. Hydrogen Production by PEM Water Electrolysis – A Review. Materials Science for Energy Technologies 2019, 2 (3), 442–454. 10.1016/j.mset.2019.03.002.

[ref5] Teuku H.; Alshami I.; Goh J.; Masdar M. S.; Loh K. S. Review on Bipolar Plates for Low-Temperature Polymer Electrolyte Membrane Water Electrolyzer. International Journal of Energy Research 2021, 45 (15), 20583–20600. 10.1002/er.7182.

[ref6] Gerhardt M. R.; Pant L. M.; Bui J. C.; Crothers A. R.; Ehlinger V. M.; Fornaciari J. C.; Liu J.; Weber A. Z. Method—Practices and Pitfalls in Voltage Breakdown Analysis of Electrochemical Energy-Conversion Systems. J. Electrochem. Soc. 2021, 168 (7), 074503 10.1149/1945-7111/abf061.

[ref7] Jackson C.; Lin X.; Levecque P. B. J.; Kucernak A. R. J. Toward Understanding the Utilization of Oxygen Reduction Electrocatalysts under High Mass Transport Conditions and High Overpotentials. ACS Catal. 2022, 12 (1), 200–211. 10.1021/acscatal.1c03908.

[ref8] Ehelebe K.; Schmitt N.; Sievers G.; Jensen A. W.; Hrnjić A.; Collantes Jiménez P.; Kaiser P.; Geuß M.; Ku Y.-P.; Jovanovič P.; Mayrhofer K. J. J.; Etzold B.; Hodnik N.; Escudero-Escribano M.; Arenz M.; Cherevko S. Benchmarking Fuel Cell Electrocatalysts Using Gas Diffusion Electrodes: Inter-Lab Comparison and Best Practices. ACS Energy Lett. 2022, 7 (2), 816–826. 10.1021/acsenergylett.1c02659.

[ref9] Ehelebe K.; Seeberger D.; Paul M. T. Y.; Thiele S.; Mayrhofer K. J. J.; Cherevko S. Evaluating Electrocatalysts at Relevant Currents in a Half-Cell: The Impact of Pt Loading on Oxygen Reduction Reaction. J. Electrochem. Soc. 2019, 166 (16), F1259 10.1149/2.0911915jes.

[ref10] Clean Hydrogen Joint Undertaking. Programme Review Report 2023; Publications Office of the European Union, 2023.

[ref11] Mao G.; Kilani M.; Ahmed M. Review—Micro/Nanoelectrodes and Their Use in Electrocrystallization: Historical Perspective and Current Trends. J. Electrochem. Soc. 2022, 169 (2), 022505 10.1149/1945-7111/ac51a0.

[ref12] Bard A. J.; Faulkner L. R.; White H. S. Electrochemical Methods: Fundamentals and Applications; John Wiley & Sons, 2022.

[ref13] Zoski C. G. Ultramicroelectrodes: Design, Fabrication, and Characterization. Electroanalysis 2002, 14 (15–16), 1041–1051. 10.1002/1521-4109(200208)14:15/16<1041::AID-ELAN1041>3.0.CO;2-8.

[ref14] Guilminot E.; Corcella A.; Chatenet M.; Maillard F. Comparing the Thin-Film Rotating Disk Electrode and the Ultramicroelectrode with Cavity Techniques to Study Carbon-Supported Platinum for Proton Exchange Membrane Fuel Cell Applications. J. Electroanal. Chem. 2007, 599 (1), 111–120. 10.1016/j.jelechem.2006.09.022.

[ref15] Strmcnik D.; Hodnik N.; Hocevar S. B.; van der Vliet D.; Zorko M.; Stamenkovic V. R.; Pihlar B.; Markovic N. M. Novel Method for Fast Characterization of High-Surface-Area Electrocatalytic Materials Using a Carbon Fiber Microelectrode. J. Phys. Chem. C 2010, 114 (6), 2640–2644. 10.1021/jp908939e.

[ref16] Chen S.; Kucernak A. Electrocatalysis under Conditions of High Mass Transport Rate: Oxygen Reduction on Single Submicrometer-Sized Pt Particles Supported on Carbon. J. Phys. Chem. B 2004, 108 (10), 3262–3276. 10.1021/jp036831j.

[ref17] Cha C. S.; Li C. M.; Yang H. X.; Liu P. F. Powder Microelectrodes. J. Electroanal. Chem. 1994, 368 (1), 47–54. 10.1016/0022-0728(93)03016-I.

[ref18] Cachet-Vivier C.; Keddam M.; Vivier V.; Yu L. T. Development of Cavity Microelectrode Devices and Their Uses in Various Research Fields. J. Electroanal. Chem. 2013, 688, 12–19. 10.1016/j.jelechem.2012.09.011.

[ref19] Li H.; Guo Y.; Jin Z. Advanced Electrochemical Techniques for Characterizing Electrocatalysis at the Single-Particle Level. Carb Neutrality 2023, 2 (1), 22 10.1007/s43979-023-00062-8.

[ref20] Antoine O.; Durand R. RRDE Study of Oxygen Reduction on Pt Nanoparticles inside Nafion®: H2O2 Production in PEMFC Cathode Conditions. J. Appl. Electrochem. 2000, 30 (7), 839–844. 10.1023/A:1003999818560.

[ref21] Lee S.-J.; Pyun S.-I.; Lee S.-K.; Kang S.-J. L. Fundamentals of Rotating Disc and Ring–Disc Electrode Techniques and Their Applications to Study of the Oxygen Reduction Mechanism at Pt/C Electrode for Fuel Cells. Isr. J. Chem. 2008, 48 (3–4), 215–228. 10.1560/IJC.48.3-4.215.

[ref22] Shinozaki K.; Zack J. W.; Richards R. M.; Pivovar B. S.; Kocha S. S. Oxygen Reduction Reaction Measurements on Platinum Electrocatalysts Utilizing Rotating Disk Electrode Technique I. Impact of Impurities, Measurement Protocols and Applied Corrections. J. Electrochem. Soc. 2015, 162 (10), F1144–F1158. 10.1149/2.1071509jes.

[ref23] Cherevko S.; Katsounaros I. And yet It Rotates!. Nat. Catal 2024, 7 (1), 10–11. 10.1038/s41929-023-01100-5.

[ref24] Schmidt T. J.; Gasteiger H. A.; Stäb G. D.; Urban P. M.; Kolb D. M.; Behm R. J. Characterization of High-Surface-Area Electrocatalysts Using a Rotating Disk Electrode Configuration. J. Electrochem. Soc. 1998, 145 (7), 2354–2358. 10.1149/1.1838642.

[ref25] Shinozaki K.; Zack J. W.; Pylypenko S.; Pivovar B. S.; Kocha S. S. Oxygen Reduction Reaction Measurements on Platinum Electrocatalysts Utilizing Rotating Disk Electrode Technique II. Influence of Ink Formulation, Catalyst Layer Uniformity and Thickness. J. Electrochem. Soc. 2015, 162 (12), F1384–F1396. 10.1149/2.0551512jes.

[ref26] Inaba M.; Quinson J.; Bucher J. R.; Arenz M. On the Preparation and Testing of Fuel Cell Catalysts Using the Thin Film Rotating Disk Electrode Method. J. Vis Exp 2018, 133, 57105 10.3791/57105. PMC5931774 29608166

[ref27] Wang R.; Jiang W.; Xia D.; Liu T.; Gan L. Improving the Wettability of Thin-Film Rotating Disk Electrodes for Reliable Activity Evaluation of Oxygen Electrocatalysts by Triggering Oxygen Reduction at the Catalyst-Electrolyte-Bubble Triple Phase Boundaries. J. Electrochem. Soc. 2018, 165 (7), F436 10.1149/2.0371807jes.

[ref28] Lazaridis T.; Stühmeier B. M.; Gasteiger H. A.; El-Sayed H. A. Capabilities and Limitations of Rotating Disk Electrodes versus Membrane Electrode Assemblies in the Investigation of Electrocatalysts. Nat. Catal 2022, 5 (5), 363–373. 10.1038/s41929-022-00776-5.

[ref29] Martens S.; Asen L.; Ercolano G.; Dionigi F.; Zalitis C.; Hawkins A.; Martinez Bonastre A.; Seidl L.; Knoll A. C.; Sharman J.; Strasser P.; Jones D.; Schneider O. A Comparison of Rotating Disc Electrode, Floating Electrode Technique and Membrane Electrode Assembly Measurements for Catalyst Testing. J. Power Sources 2018, 392, 274–284. 10.1016/j.jpowsour.2018.04.084.

[ref30] Temmel S. E.; Tschupp S. A.; Schmidt T. J. A Highly Flexible Electrochemical Flow Cell Designed for the Use of Model Electrode Materials on Non-Conventional Substrates. Rev. Sci. Instrum. 2016, 87 (4), 045115 10.1063/1.4947459. 27131715

[ref31] Wang H.; Jusys Z.; Behm R. J.; Abruña H. D. A Channel Flow Cell with Double Disk Electrodes for Oxygen Electroreduction Study at Elevated Temperatures and Pressures: Theory. J. Electroanal. Chem. 2021, 896, 115251 10.1016/j.jelechem.2021.115251.

[ref32] Zalitis C. M.; Kramer D.; Kucernak A. R. Electrocatalytic Performance of Fuel Cell Reactions at Low Catalyst Loading and High Mass Transport. Phys. Chem. Chem. Phys. 2013, 15 (12), 4329–4340. 10.1039/c3cp44431g. 23407648

[ref33] Lin X.; Zalitis C. M.; Sharman J.; Kucernak A. Electrocatalyst Performance at the Gas/Electrolyte Interface under High-Mass-Transport Conditions: Optimization of the “Floating Electrode” Method. ACS Appl. Mater. Interfaces 2020, 12 (42), 47467–47481. 10.1021/acsami.0c12718. 32986947

[ref34] Jackson C.; Raymakers L. F. J. M.; Mulder M. J. J.; Kucernak A. R. J. Assessing Electrocatalyst Hydrogen Activity and CO Tolerance: Comparison of Performance Obtained Using the High Mass Transport ‘Floating Electrode’ Technique and in Electrochemical Hydrogen Pumps. Applied Catalysis B: Environmental 2020, 268, 118734 10.1016/j.apcatb.2020.118734.

[ref35] Markiewicz M.; Zalitis C.; Kucernak A. Performance Measurements and Modelling of the ORR on Fuel Cell Electrocatalysts – the Modified Double Trap Model. Electrochim. Acta 2015, 179, 126–136. 10.1016/j.electacta.2015.04.066.

[ref36] Zalitis C. M.; Sharman J.; Wright E.; Kucernak A. R. Properties of the Hydrogen Oxidation Reaction on Pt/C Catalysts at Optimised High Mass Transport Conditions and Its Relevance to the Anode Reaction in PEFCs and Cathode Reactions in Electrolysers. Electrochim. Acta 2015, 176, 763–776. 10.1016/j.electacta.2015.06.146.

[ref37] Suter T. A. M.; Smith K.; Hack J.; Rasha L.; Rana Z.; Angel G. M. A.; Shearing P. R.; Miller T. S.; Brett D. J. L. Engineering Catalyst Layers for Next-Generation Polymer Electrolyte Fuel Cells: A Review of Design, Materials, and Methods. Adv. Energy Mater. 2021, 11 (37), 2101025 10.1002/aenm.202101025.

[ref38] He W.; Nguyen T. V. Edge Effects on Reference Electrode Measurements in PEM Fuel Cells. J. Electrochem. Soc. 2004, 151 (2), A185 10.1149/1.1634272.

[ref39] Nogueira J. A.; Krischer K.; Varela H. Coupled Dynamics of Anode and Cathode in Proton-Exchange Membrane Fuel Cells. ChemPhysChem 2019, 20 (22), 3081–3088. 10.1002/cphc.201900531. 31322819

[ref40] Rabissi C.; Brightman E.; Hinds G.; Casalegno A. In Operando Investigation of Anode Overpotential Dynamics in Direct Methanol Fuel Cells. Int. J. Hydrogen Energy 2016, 41 (40), 18221–18225. 10.1016/j.ijhydene.2016.08.140.

[ref41] Uhm S.; Chung S. T.; Lee J. Characterization of Direct Formic Acid Fuel Cells by Impedance Studies: In Comparison of Direct Methanol Fuel Cells. J. Power Sources 2008, 178 (1), 34–43. 10.1016/j.jpowsour.2007.12.016.

[ref42] Inaba M.; Jensen A. W.; Sievers G. W.; Escudero-Escribano M.; Zana A.; Arenz M. Benchmarking High Surface Area Electrocatalysts in a Gas Diffusion Electrode: Measurement of Oxygen Reduction Activities under Realistic Conditions. Energy Environ. Sci. 2018, 11 (4), 988–994. 10.1039/C8EE00019K.

[ref43] Chen Y.-X.; Li M.-F.; Liao L.-W.; Xu J.; Ye S. A Thermostatic Cell with Gas Diffusion Electrode for Oxygen Reduction Reaction under Fuel Cell Relevant Conditions. Electrochem. Commun. 2009, 11 (7), 1434–1436. 10.1016/j.elecom.2009.05.023.

[ref44] Alinejad S.; Inaba M.; Schröder J.; Du J.; Quinson J.; Zana A.; Arenz M. Testing Fuel Cell Catalysts under More Realistic Reaction Conditions: Accelerated Stress Tests in a Gas Diffusion Electrode Setup. J. Phys. Energy 2020, 2 (2), 024003 10.1088/2515-7655/ab67e2.

[ref45] Sievers G. W.; Jensen A. W.; Brüser V.; Arenz M.; Escudero-Escribano M. Sputtered Platinum Thin-Films for Oxygen Reduction in Gas Diffusion Electrodes: A Model System for Studies under Realistic Reaction Conditions. Surfaces 2019, 2 (2), 336–348. 10.3390/surfaces2020025.

[ref46] Pinaud B. A.; Bonakdarpour A.; Daniel L.; Sharman J.; Wilkinson D. P. Key Considerations for High Current Fuel Cell Catalyst Testing in an Electrochemical Half-Cell. J. Electrochem. Soc. 2017, 164 (4), F321 10.1149/2.0891704jes.

[ref47] Ehelebe K.; Knöppel J.; Bierling M.; Mayerhöfer B.; Böhm T.; Kulyk N.; Thiele S.; Mayrhofer K. J. J.; Cherevko S. Platinum Dissolution in Realistic Fuel Cell Catalyst Layers. Angew. Chem., Int. Ed. 2021, 60 (16), 8882–8888. 10.1002/anie.202014711. PMC8048487 33410273

[ref48] Hrnjić A.; Ruiz-Zepeda F.; Gaberšček M.; Bele M.; Suhadolnik L.; Hodnik N.; Jovanovič P. Modified Floating Electrode Apparatus for Advanced Characterization of Oxygen Reduction Reaction Electrocatalysts. J. Electrochem. Soc. 2020, 167 (16), 166501 10.1149/1945-7111/abc9de.

[ref49] Bele M.; Podboršek G. K.; Lončar A.; Jovanovič P.; Hrnjić A.; Marinko Ž.; Kovač J.; Surca A. K.; Kamšek A. R.; Dražić G.; Hodnik N.; Suhadolnik L. Nano Lab” Advanced Characterization Platform for Studying Electrocatalytic Iridium Nanoparticles Dispersed on TiOxNy Supports Prepared on Ti Transmission Electron Microscopy Grids. ACS Appl. Nano Mater. 2023, 6 (12), 10421–10430. 10.1021/acsanm.3c01368. 37384128 PMC10294127

[ref50] Schmitt N.; Schmidt M.; Hübner G.; Etzold B. J. M. Oxygen Reduction Reaction Measurements on Platinum Electrocatalysts in Gas Diffusion Electrode Half-Cells: Influence of Electrode Preparation, Measurement Protocols and Common Pitfalls. J. Power Sources 2022, 539, 231530 10.1016/j.jpowsour.2022.231530.

[ref51] Schmitt N.; Schmidt M.; Mueller J. E.; Schmidt L.; Trabold M.; Jeschonek K.; Etzold B. J. M. Which Insights Can Gas Diffusion Electrode Half-Cell Experiments Give into Activity Trends and Transport Phenomena of Membrane Electrode Assemblies?. Energy Adv. 2023, 2 (6), 854–863. 10.1039/D3YA00055A.

[ref52] Loukrakpam R.; Gomes B. F.; Kottakkat T.; Roth C. A Bird’s Eye Perspective of the Measurement of Oxygen Reduction Reaction in Gas Diffusion Electrode Half-Cell Set-Ups for Pt Electrocatalysts in Acidic Media. J. Phys. Mater. 2021, 4 (4), 044004 10.1088/2515-7639/ac0319.

[ref53] Nösberger S.; Du J.; Quinson J.; Berner E.; Zana A.; Wiberg G. K. H.; Arenz M. The Gas Diffusion Electrode Setup as a Testing Platform for Evaluating Fuel Cell Catalysts: A Comparative RDE-GDE Study. Electrochemical Science Advances 2023, 3 (1), e2100190 10.1002/elsa.202100190.

[ref54] Parthasarathy A.; Martin C. R.; Srinivasan S. Investigations of the O 2 Reduction Reaction at the Platinum/Nafion® Interface Using a Solid-State Electrochemical Cell. J. Electrochem. Soc. 1991, 138 (4), 916 10.1149/1.2085747.

[ref55] Parthasarathy A.; Davé B.; Srinivasan S.; Appleby A. J.; Martin C. R. The Platinum Microelectrode/Nafion Interface: An Electrochemical Impedance Spectroscopic Analysis of Oxygen Reduction Kinetics and Nafion Characteristics. J. Electrochem. Soc. 1992, 139 (6), 1634 10.1149/1.2069469.

[ref56] Srinivasan S.; Velev O. A.; Parthasarathy A.; Manko D. J.; Appleby A. J. High Energy Efficiency and High Power Density Proton Exchange Membrane Fuel Cells — Electrode Kinetics and Mass Transport. J. Power Sources 1991, 36 (3), 299–320. 10.1016/0378-7753(91)87009-Z.

[ref57] Basura V.; Beattie P. D.; Holdcroft S. Solid-State Electrochemical Oxygen Reduction at Pt | Nafion® 117 and Pt | BAM3G 407 Interfaces. J. Electroanal. Chem. 1998, 458 (1), 1–5. 10.1016/S0022-0728(98)00338-6.

[ref58] Beattie P. D.; Basura V. I.; Holdcroft S. Temperature and Pressure Dependence of O2 Reduction at Pt | Nafion® 117 and Pt | BAM® 407 Interfaces. J. Electroanal. Chem. 1999, 468 (2), 180–192. 10.1016/S0022-0728(99)00164-3.

[ref59] Petrovick J. G.; Anderson G. C.; Kushner D. I.; Danilovic N.; Weber A. Z. Method—Using Microelectrodes to Explore Solid Polymer Electrolytes. J. Electrochem. Soc. 2021, 168 (5), 056517 10.1149/1945-7111/abee5f.

[ref60] Wang J. X.; Zhang J.; Adzic R. R. Double-Trap Kinetic Equation for the Oxygen Reduction Reaction on Pt(111) in Acidic Media. J. Phys. Chem. A 2007, 111 (49), 12702–12710. 10.1021/jp076104e. 18052309

[ref61] Compton R. G.; Sokolov S. V. Electrochemistry Needs Electrochemists: “Goodbye to Rotating Discs. J. Solid State Electrochem 2024, 28 (3), 1041–1047. 10.1007/s10008-023-05443-8.

[ref62] Zalitis C.; Kucernak A.; Lin X.; Sharman J. Electrochemical Measurement of Intrinsic Oxygen Reduction Reaction Activity at High Current Densities as a Function of Particle Size for Pt4–xCox/C (x = 0, 1, 3) Catalysts. ACS Catal. 2020, 10 (7), 4361–4376. 10.1021/acscatal.9b04750.

[ref63] Zheng J.; Yan Y.; Xu B. Correcting the Hydrogen Diffusion Limitation in Rotating Disk Electrode Measurements of Hydrogen Evolution Reaction Kinetics. J. Electrochem. Soc. 2015, 162 (14), F1470 10.1149/2.0501514jes.

[ref64] Marković N. M.; Gasteiger H. A.; Grgur B. N.; Ross P. N. Oxygen Reduction Reaction on Pt(111): Effects of Bromide. J. Electroanal. Chem. 1999, 467 (1), 157–163. 10.1016/S0022-0728(99)00020-0.

[ref65] Lide D. R. CRC Handbook of Chemistry and Physics, 85th ed.; CRC Press, 2004.

[ref66] Reshetenko T. V.; St-Pierre J. Separation Method for Oxygen Mass Transport Coefficient in Gas and Ionomer Phases in PEMFC GDE. J. Electrochem. Soc. 2014, 161 (10), F1089 10.1149/2.1021410jes.

[ref67] Zalitis C. M.; Kramer D.; Sharman J.; Wright E.; Kucernak A. R. Pt Nano-Particle Performance for PEFC Reactions at Low Catalyst Loading and High Reactant Mass Transport. ECS Trans. 2013, 58 (1), 39 10.1149/05801.0039ecst.

[ref68] Jinnouchi R.; Kudo K.; Kodama K.; Kitano N.; Suzuki T.; Minami S.; Shinozaki K.; Hasegawa N.; Shinohara A. The Role of Oxygen-Permeable Ionomer for Polymer Electrolyte Fuel Cells. Nat. Commun. 2021, 12 (1), 4956 10.1038/s41467-021-25301-3. 34400643 PMC8368003

[ref69] Shinozaki K.; Kajiya S.; Yamakawa S.; Hasegawa N.; Suzuki T.; Shibata M.; Jinnouchi R. Investigation of Gas Transport Resistance in Fuel Cell Catalyst Layers via Hydrogen Limiting Current Measurements of CO-Covered Catalyst Surfaces. J. Power Sources 2023, 565, 232909 10.1016/j.jpowsour.2023.232909.

[ref70] Lauf P.; Lloret V.; Geuß M.; Collados C. C.; Thommes M.; Mayrhofer K. J. J.; Ehelebe K.; Cherevko S. Characterization of Oxygen and Ion Mass Transport Resistance in Fuel Cell Catalyst Layers in Gas Diffusion Electrode Setups. J. Electrochem. Soc. 2023, 170 (6), 064509 10.1149/1945-7111/acdafb.

[ref71] Myland J. C.; Oldham K. B. Uncompensated Resistance. 1. The Effect of Cell Geometry. Anal. Chem. 2000, 72 (17), 3972–3980. 10.1021/ac0001535. 10994953

[ref72] Newman J.; Balsara N. Applications of Potential Theory. In Electrochemical Systems; John Wiley & Sons, 2021; pp 365–398.

[ref73] Pletcher D.; Greff R.; Peat R.; Peter L. M.; Robinson J. The Design of Electrochemical Experiments. In Instrumental Methods in Electrochemistry; Woodhead Publishing, 2011; pp 356–388.

[ref74] Vesztergom S.; Barankai N.; Kovács N.; Ujvári M.; Broekmann P.; Siegenthaler H.; Láng G. G. Electrical Cross-Talk in Rotating Ring–Disk Experiments. Electrochem. Commun. 2016, 68, 54–58. 10.1016/j.elecom.2016.04.012.

[ref75] Vesztergom S. Rotating Disk and Ring–Disk Electrodes. In Encyclopedia of Interfacial Chemistry; Elsevier, 2018; pp 421–444. 10.1016/B978-0-12-409547-2.13563-2.

[ref76] Wiberg G. K. H.; Nösberger S.; Arenz M. Evolution of a GDE Setup: Beyond Ambient Conditions. Current Opinion in Electrochemistry 2022, 36, 101129 10.1016/j.coelec.2022.101129.

[ref77] Kulikovsky A. A.; Berg P. Positioning of a Reference Electrode in a PEM Fuel Cell. J. Electrochem. Soc. 2015, 162 (8), F843 10.1149/2.0231508jes.

[ref78] Fleige M. J.; Wiberg G. K. H.; Arenz M. Rotating Disk Electrode System for Elevated Pressures and Temperatures. Rev. Sci. Instrum. 2015, 86 (6), 064101 10.1063/1.4922382. 26133849

[ref79] Kucernak A. R.; Zalitis C. General Models for the Electrochemical Hydrogen Oxidation and Hydrogen Evolution Reactions: Theoretical Derivation and Experimental Results under Near Mass-Transport Free Conditions. J. Phys. Chem. C 2016, 120 (20), 10721–10745. 10.1021/acs.jpcc.6b00011.

[ref80] Wiberg G. K. H.; Fleige M.; Arenz M. Gas Diffusion Electrode Setup for Catalyst Testing in Concentrated Phosphoric Acid at Elevated Temperatures. Rev. Sci. Instrum. 2015, 86 (2), 024102 10.1063/1.4908169. 25725862

[ref81] Katsounaros I.; Meier J. C.; Klemm S. O.; Topalov A. A.; Biedermann P. U.; Auinger M.; Mayrhofer K. J. J. The Effective Surface pH during Reactions at the Solid–Liquid Interface. Electrochem. Commun. 2011, 13 (6), 634–637. 10.1016/j.elecom.2011.03.032.

[ref82] He Q.; Yang X.; Chen W.; Mukerjee S.; Koel B.; Chen S. Influence of Phosphate Anion Adsorption on the Kinetics of Oxygen Electroreduction on Low Index Pt(Hkl) Single Crystals. Phys. Chem. Chem. Phys. 2010, 12 (39), 12544–12555. 10.1039/c0cp00433b. 20725683

[ref83] Lee C.-Y.; Chuang S.-M.; Lee S.-J.; Chiu C.-Y. Fabrication of Flexible Micro pH Sensor for Use in Proton Exchange Membrane Fuel Cell. Int. J. Electrochem. Sci. 2016, 11 (3), 2263–2268. 10.1016/S1452-3981(23)16099-8.

[ref84] Yarlagadda V.; McKinney S. E.; Keary C. L.; Thompson L.; Zulevi B.; Kongkanand A. Preparation of PEMFC Electrodes from Milligram-Amounts of Catalyst Powder. J. Electrochem. Soc. 2017, 164 (7), F845 10.1149/2.1461707jes.

[ref85] Jackson C.; Metaxas M.; Dawson J.; Kucernak A. R. Nanostructured Catalyst Layer Allowing Production of Ultralow Loading Electrodes for Polymer Electrolyte Membrane Fuel Cells with Superior Performance. ACS Appl. Energy Mater. 2023, 6 (24), 12296–12306. 10.1021/acsaem.3c01987. 38155874 PMC10751738

[ref86] Ke K.; Hiroshima K.; Kamitaka Y.; Hatanaka T.; Morimoto Y. An Accurate Evaluation for the Activity of Nano-Sized Electrocatalysts by a Thin-Film Rotating Disk Electrode: Oxygen Reduction on Pt/C. Electrochim. Acta 2012, 72, 120–128. 10.1016/j.electacta.2012.04.004.

[ref87] Shi Z.; Zhang X.; Lin X.; Liu G.; Ling C.; Xi S.; Chen B.; Ge Y.; Tan C.; Lai Z.; Huang Z.; Ruan X.; Zhai L.; Li L.; Li Z.; Wang X.; Nam G.-H.; Liu J.; He Q.; Guan Z.; Wang J.; Lee C.-S.; Kucernak A. R. J.; Zhang H. Phase-Dependent Growth of Pt on MoS2 for Highly Efficient H2 Evolution. Nature 2023, 621 (7978), 300–305. 10.1038/s41586-023-06339-3. 37704763

[ref88] Green C. L.; Kucernak A. Determination of the Platinum and Ruthenium Surface Areas in Platinum–Ruthenium Alloy Electrocatalysts by Underpotential Deposition of Copper. I. Unsupported Catalysts. J. Phys. Chem. B 2002, 106 (5), 1036–1047. 10.1021/jp0131931.

[ref89] Röttcher N. C.; Ku Y.-P.; Minichova M.; Ehelebe K.; Cherevko S. Comparison of Methods to Determine Electrocatalysts’ Surface Area in Gas Diffusion Electrode Setups: A Case Study on Pt/C and PtRu/C. J. Phys. Energy 2023, 5 (2), 024007 10.1088/2515-7655/acbe1b.

[ref90] Yano H.; Uematsu T.; Omura J.; Watanabe M.; Uchida H. Effect of Adsorption of Sulfate Anions on the Activities for Oxygen Reduction Reaction on Nafion®-Coated Pt/Carbon Black Catalysts at Practical Temperatures. J. Electroanal. Chem. 2015, 747, 91–96. 10.1016/j.jelechem.2015.04.007.

[ref91] McCrory C. C. L.; Jung S.; Peters J. C.; Jaramillo T. F. Benchmarking Heterogeneous Electrocatalysts for the Oxygen Evolution Reaction. J. Am. Chem. Soc. 2013, 135 (45), 16977–16987. 10.1021/ja407115p. 24171402

[ref92] Shinozaki K.; Morimoto Y.; Pivovar B. S.; Kocha S. S. Re-Examination of the Pt Particle Size Effect on the Oxygen Reduction Reaction for Ultrathin Uniform Pt/C Catalyst Layers without Influence from Nafion. Electrochim. Acta 2016, 213, 783–790. 10.1016/j.electacta.2016.08.001.

[ref93] Hodnik N.; Baldizzone C.; Cherevko S.; Zeradjanin A.; Mayrhofer K. J. J. The Effect of the Voltage Scan Rate on the Determination of the Oxygen Reduction Activity of Pt/C Fuel Cell Catalyst. Electrocatalysis 2015, 6 (3), 237–241. 10.1007/s12678-015-0255-0.

[ref94] Zalitis C. M.; Kucernak A. R.; Sharman J.; Wright E. Design Principles for Platinum Nanoparticles Catalysing Electrochemical Hydrogen Evolution and Oxidation Reactions: Edges Are Much More Active than Facets. J. Mater. Chem. A 2017, 5 (44), 23328–23338. 10.1039/C7TA05543A.

[ref95] Koutecky J.; Levich V. The Use of a Rotating Disk Electrode in the Studies of Electrochemical Kinetics and Electrolytic Processes. Zh. Fiz. Khim. 1958, 32, 1565–1575.

[ref96] Levich V. Physicochemical Hydrodynamics; University of California, Prentice-Hall, 1962.

[ref97] Wang J. X.; Uribe F. A.; Springer T. E.; Zhang J.; Adzic R. R. Intrinsic Kinetic Equation for Oxygen Reduction Reaction in Acidic Media: The Double Tafel Slope and Fuel Cell Applications. Faraday Discuss. 2009, 140, 347–362. 10.1039/B802218F. 19213326

[ref98] Fan J.; Chen M.; Zhao Z.; Zhang Z.; Ye S.; Xu S.; Wang H.; Li H. Bridging the Gap between Highly Active Oxygen Reduction Reaction Catalysts and Effective Catalyst Layers for Proton Exchange Membrane Fuel Cells. Nat. Energy 2021, 6 (5), 475–486. 10.1038/s41560-021-00824-7.

[ref99] Pan L.; Ott S.; Dionigi F.; Strasser P. Current Challenges Related to the Deployment of Shape-Controlled Pt Alloy Oxygen Reduction Reaction Nanocatalysts into Low Pt-Loaded Cathode Layers of Proton Exchange Membrane Fuel Cells. Current Opinion in Electrochemistry 2019, 18, 61–71. 10.1016/j.coelec.2019.10.011.

[ref100] Schröder J.; Mints V. A.; Bornet A.; Berner E.; Fathi Tovini M.; Quinson J.; Wiberg G. K. H.; Bizzotto F.; El-Sayed H. A.; Arenz M. The Gas Diffusion Electrode Setup as Straightforward Testing Device for Proton Exchange Membrane Water Electrolyzer Catalysts. JACS Au 2021, 1 (3), 247–251. 10.1021/jacsau.1c00015. 34467289 PMC8395656

[ref101] Jovanovič P.; Stojanovski K.; Bele M.; Dražić G.; Koderman Podboršek G.; Suhadolnik L.; Gaberšček M.; Hodnik N. Methodology for Investigating Electrochemical Gas Evolution Reactions: Floating Electrode as a Means for Effective Gas Bubble Removal. Anal. Chem. 2019, 91 (16), 10353–10356. 10.1021/acs.analchem.9b01317. 31379155 PMC6748558

[ref102] Sherwin C.; Celorrio V.; Podbevsek U.; Rigg K.; Hodges T.; Ibraliu A.; Telfer A.; McLeod L.; Difilippo A.; Corbos E. C.; Zalitis C.; Russell A. E. An Optimised Cell for in Situ XAS of Gas Diffusion Electrocatalyst Electrodes. ChemCatChem 2024, e202400221 10.1002/cctc.202400221.

[ref103] Geuß M.; Milosevic M.; Bierling M.; Löttert L.; Abbas D.; Escalera-López D.; Lloret V.; Ehelebe K.; Mayrhofer K. J. J.; Thiele S.; Cherevko S. Investigation of Iridium-Based OER Catalyst Layers in a GDE Half-Cell Setup: Opportunities and Challenges. J. Electrochem. Soc. 2023, 170 (11), 114510 10.1149/1945-7111/ad07ac.

[ref104] Ripatti D. S.; Veltman T. R.; Kanan M. W. Carbon Monoxide Gas Diffusion Electrolysis That Produces Concentrated C2 Products with High Single-Pass Conversion. Joule 2019, 3 (1), 240–256. 10.1016/j.joule.2018.10.007.

[ref105] Han L.; Zhou W.; Xiang C. High-Rate Electrochemical Reduction of Carbon Monoxide to Ethylene Using Cu-Nanoparticle-Based Gas Diffusion Electrodes. ACS Energy Lett. 2018, 3 (4), 855–860. 10.1021/acsenergylett.8b00164.

[ref106] Hasa B.; Jouny M.; Ko B. H.; Xu B.; Jiao F. Flow Electrolyzer Mass Spectrometry with a Gas-Diffusion Electrode Design. Angew. Chem., Int. Ed. 2021, 60 (6), 3277–3282. 10.1002/anie.202013713. 33090694

[ref107] Kopljar D.; Inan A.; Vindayer P.; Wagner N.; Klemm E. Electrochemical Reduction of CO2 to Formate at High Current Density Using Gas Diffusion Electrodes. J. Appl. Electrochem. 2014, 44 (10), 1107–1116. 10.1007/s10800-014-0731-x.

[ref108] Lees E. W.; Mowbray B. A. W.; Parlane F. G. L.; Berlinguette C. P. Gas Diffusion Electrodes and Membranes for CO2 Reduction Electrolysers. Nat. Rev. Mater. 2022, 7 (1), 55–64. 10.1038/s41578-021-00356-2.

[ref109] Zhang G.; Kucernak A. Gas Accessible Membrane Electrode (GAME): A Versatile Platform for Elucidating Electrocatalytic Processes Using Real-Time and in Situ Hyphenated Electrochemical Techniques. ACS Catal. 2020, 10 (17), 9684–9693. 10.1021/acscatal.0c02433.

[ref110] Zhang G.; Cui Y.; Kucernak A. Real-Time In Situ Monitoring of CO2 Electroreduction in the Liquid and Gas Phases by Coupled Mass Spectrometry and Localized Electrochemistry. ACS Catal. 2022, 12 (10), 6180–6190. 10.1021/acscatal.2c00609. 35633901 PMC9127967

[ref111] de Jesus Gálvez-Vázquez M.; Moreno-García P.; Xu H.; Hou Y.; Hu H.; Montiel I. Z.; Rudnev A. V.; Alinejad S.; Grozovski V.; Wiley B. J.; Arenz M.; Broekmann P. Environment Matters: CO2RR Electrocatalyst Performance Testing in a Gas-Fed Zero-Gap Electrolyzer. ACS Catal. 2020, 10 (21), 13096–13108. 10.1021/acscatal.0c03609.

[ref112] Zhang L.; Zhang J. Novel Electrochemical Half-Cell Design and Fabrication for Performance Analysis of Metal-Air Battery Air-Cathodes. International Journal of Green Energy 2019, 16 (3), 236–241. 10.1080/15435075.2018.1555760.

[ref113] Davidson E. A.; Winiwarter W. Urgent Abatement of Industrial Sources of Nitrous Oxide. Nat. Clim. Chang. 2023, 13 (7), 599–601. 10.1038/s41558-023-01723-3.

[ref114] Erickson L. E.; Newmark G. L.; Higgins M. J.; Wang Z. Nitrogen Oxides and Ozone in Urban Air: A Review of 50 plus Years of Progress. Environmental Progress & Sustainable Energy 2020, 39 (6), e13484 10.1002/ep.13484.

[ref115] Gridin V.; Du J.; Haller S.; Theis P.; Hofmann K.; Wiberg G. K. H.; Kramm U. I.; Arenz M. GDE vs RDE: Impact of Operation Conditions on Intrinsic Catalytic Parameters of FeNC Catalyst for the Oxygen Reduction Reaction. Electrochim. Acta 2023, 444, 142012 10.1016/j.electacta.2023.142012.

[ref116] Teppor P.; Jäger R.; Koppel M.; Volobujeva O.; Palm R.; Månsson M.; Härk E.; Kochovski Z.; Aruväli J.; Kooser K.; Granroth S.; Käämbre T.; Nerut J.; Lust E. Unlocking the Porosity of Fe–N–C Catalysts Using Hydroxyapatite as a Hard Template En Route to Eco-Friendly High-Performance AEMFCs. J. Power Sources 2024, 591, 233816 10.1016/j.jpowsour.2023.233816.

[ref117] Bondue C. J.; Abd-El-Latif A. A.; Hegemann P.; Baltruschat H. Quantitative Study for Oxygen Reduction and Evolution in Aprotic Organic Electrolytes at Gas Diffusion Electrodes by DEMS. J. Electrochem. Soc. 2015, 162 (3), A479 10.1149/2.0871503jes.

[ref118] Li F.; Mocci F.; Zhang X.; Ji X.; Laaksonen A. Ionic Liquids for CO2 Electrochemical Reduction. Chinese Journal of Chemical Engineering 2021, 31, 75–93. 10.1016/j.cjche.2020.10.029.

[ref119] Lasia A. Electrochemical Impedance Spectroscopy and Its Applications; Springer, 2014.

[ref120] Orazem M. E.; Tribollet B. Electrochemical Impedance Spectroscopy, 2nd ed.; John Wiley & Sons, 2017.

[ref121] Ku Y.-P.; Kumar K.; Hutzler A.; Götz C.; Vorochta M.; Sougrati M. T.; Lloret V.; Ehelebe K.; Mayrhofer K. J. J.; Thiele S.; Khalakhan I.; Böhm T.; Jaouen F.; Cherevko S. Impact of Carbon Corrosion and Denitrogenation on the Deactivation of Fe–N–C Catalysts in Alkaline Media. ACS Catal. 2024, 14 (11), 8576–8591. 10.1021/acscatal.4c01219.

[ref122] Ehelebe K.; Escalera-López D.; Cherevko S. Limitations of Aqueous Model Systems in the Stability Assessment of Electrocatalysts for Oxygen Reactions in Fuel Cell and Electrolyzers. Current Opinion in Electrochemistry 2021, 29, 100832 10.1016/j.coelec.2021.100832.

[ref123] Barham J. P.; König B. Synthetic Photoelectrochemistry. Angew. Chem., Int. Ed. 2020, 59 (29), 11732–11747. 10.1002/anie.201913767. PMC7383880 31805216

[ref124] Ahmed M.; Dincer I. A Review on Photoelectrochemical Hydrogen Production Systems: Challenges and Future Directions. Int. J. Hydrogen Energy 2019, 44 (5), 2474–2507. 10.1016/j.ijhydene.2018.12.037.

[ref125] Lozeman J. J. A.; Führer P.; Olthuis W.; Odijk M. Spectroelectrochemistry, the Future of Visualizing Electrode Processes by Hyphenating Electrochemistry with Spectroscopic Techniques. Analyst 2020, 145 (7), 2482–2509. 10.1039/C9AN02105A. 31998878

[ref126] Zhao E. W.; Liu T.; Jónsson E.; Lee J.; Temprano I.; Jethwa R. B.; Wang A.; Smith H.; Carretero-González J.; Song Q.; Grey C. P. In Situ NMR Metrology Reveals Reaction Mechanisms in Redox Flow Batteries. Nature 2020, 579 (7798), 224–228. 10.1038/s41586-020-2081-7. 32123353

[ref127] Kutin Y.; Cox N.; Lubitz W.; Schnegg A.; Rüdiger O. In Situ EPR Characterization of a Cobalt Oxide Water Oxidation Catalyst at Neutral pH. Catalysts 2019, 9 (11), 926 10.3390/catal9110926.

[ref128] Grozovski V.; Vesztergom S.; Láng G. G.; Broekmann P. Electrochemical Hydrogen Evolution: H + or H 2 O Reduction? A Rotating Disk Electrode Study. J. Electrochem. Soc. 2017, 164 (11), E3171–E3178. 10.1149/2.0191711jes.

[ref129] Hodnik N.; Cherevko S. Spot the Difference at the Nanoscale: Identical Location Electron Microscopy in Electrocatalysis. Current Opinion in Electrochemistry 2019, 15, 73–82. 10.1016/j.coelec.2019.03.007.

[ref130] Đukić T.; Pavko L.; Jovanovič P.; Maselj N.; Gatalo M.; Hodnik N. Stability Challenges of Carbon-Supported Pt-Nanoalloys as Fuel Cell Oxygen Reduction Reaction Electrocatalysts. Chem. Commun. 2022, 58 (100), 13832–13854. 10.1039/D2CC05377B. PMC9753161 36472187

[ref131] Moriau L. J.; Hrnjić A.; Pavlišič A.; Kamšek A. R.; Petek U.; Ruiz-Zepeda F.; Šala M.; Pavko L.; Šelih V. S.; Bele M.; Jovanovič P.; Gatalo M.; Hodnik N. Resolving the Nanoparticles’ Structure-Property Relationships at the Atomic Level: A Study of Pt-Based Electrocatalysts. iScience 2021, 24 (2), 102102 10.1016/j.isci.2021.102102. 33659872 PMC7890412

[ref132] Schröder J.; Quinson J.; Mathiesen J. K.; Kirkensgaard J. J. K.; Alinejad S.; Mints V. A.; Jensen K. M.; Arenz M. A New Approach to Probe the Degradation of Fuel Cell Catalysts under Realistic Conditions: Combining Tests in a Gas Diffusion Electrode Setup with Small Angle X-Ray Scattering. J. Electrochem. Soc. 2020, 167 (13), 134515 10.1149/1945-7111/abbdd2.

[ref133] Gebhard M.; Paulisch M.; Hilger A.; Franzen D.; Ellendorff B.; Turek T.; Manke I.; Roth C. Design of an In-Operando Cell for X-Ray and Neutron Imaging of Oxygen-Depolarized Cathodes in Chlor-Alkali Electrolysis. Materials 2019, 12 (8), 1275 10.3390/ma12081275. 31003446 PMC6514560

